# Syntrophic bacterial and host–microbe interactions in bacterial vaginosis

**DOI:** 10.1093/ismejo/wraf055

**Published:** 2025-06-27

**Authors:** Elliot M Lee, Sujatha Srinivasan, Samuel O Purvine, Tina L Fiedler, Owen P Leiser, Sean C Proll, Samuel S Minot, Danijel Djukovic, Daniel Raftery, Christine Johnston, David N Fredricks, Brooke L Deatherage Kaiser

**Affiliations:** Vaccine and Infectious Disease Division, Fred Hutchinson Cancer Center, 1100 Fairview Ave N Seattle, WA 98109, United States; Vaccine and Infectious Disease Division, Fred Hutchinson Cancer Center, 1100 Fairview Ave N Seattle, WA 98109, United States; Environmental Molecular Sciences Division, Pacific Northwest National Laboratory, 3335 Innovation Blvd, Richland, WA 99354, United States; Vaccine and Infectious Disease Division, Fred Hutchinson Cancer Center, 1100 Fairview Ave N Seattle, WA 98109, United States; Chemical and Biological Signatures, Pacific Northwest National Laboratory, 1100 Dexter Avenue N, Seattle, WA 98109, United States; Vaccine and Infectious Disease Division, Fred Hutchinson Cancer Center, 1100 Fairview Ave N Seattle, WA 98109, United States; Vaccine and Infectious Disease Division, Fred Hutchinson Cancer Center, 1100 Fairview Ave N Seattle, WA 98109, United States; Department of Anesthesiology and Pain Medicine, University of Washington, 1959 NE Pacific Street, Seattle, WA 98195, United States; Department of Anesthesiology and Pain Medicine, University of Washington, 1959 NE Pacific Street, Seattle, WA 98195, United States; Vaccine and Infectious Disease Division, Fred Hutchinson Cancer Center, 1100 Fairview Ave N Seattle, WA 98109, United States; Department of Medicine, University of Washington, 1959 NE Pacific Street, Seattle, WA 98195, United States; Vaccine and Infectious Disease Division, Fred Hutchinson Cancer Center, 1100 Fairview Ave N Seattle, WA 98109, United States; Department of Medicine, University of Washington, 1959 NE Pacific Street, Seattle, WA 98195, United States; Chemical and Biological Signatures, Pacific Northwest National Laboratory, 1100 Dexter Avenue N, Seattle, WA 98109, United States

**Keywords:** bacterial vaginosis, metaproteomics, microbiome, syntrophy, host–microbe interactions, microbial ecology, metabolism, fermentation, drug targets

## Abstract

Bacterial vaginosis (BV) is a common, polymicrobial condition of the vaginal microbiota that is associated with symptoms such as malodor and excessive discharge, along with increased risk of various adverse sequelae. Host–bacteria and bacteria–bacteria interactions are thought to contribute to the condition, but many of these functions have yet to be elucidated. Using untargeted metaproteomics, we identified 1068 host and 1418 bacterial proteins in a set of cervicovaginal lavage samples collected from 20 participants with BV and 9 who were negative for the condition. We identified *Dialister micraerophilus* as a major producer of malodorous polyamines and identified a syntrophic interaction between this organism and *Fannyhessea vaginae* that leads to increased production of putrescine, a metabolite characteristic of BV. Although formate synthesis has not previously been noted in BV, we discovered diverse bacteria associated with the condition express pyruvate formate-lyase enzymes *in vivo* and confirm these organisms secrete formic acid *in vitro*. Sodium hypophosphite efficiently inhibited this function in multiple taxa. We also found that the fastidious organism *Coriobacteriales* bacterium DNF00809 can metabolize formic acid secreted by *Gardnerella vaginalis*, representing another syntrophic interaction. We noted an increased abundance of the host epithelial repair protein transglutaminase 3 in the metaproteomic data, which we confirmed by enzyme-linked immunosorbent assay. Other proteins identified in our samples implicate *Finegoldia magna* and *Parvimonas micra* in the production of malodorous trimethylamine. Some bacterial proteins identified represent novel targets for future therapeutics to disrupt BV communities and promote vaginal colonization by commensal lactobacilli.

## Introduction

An optimal vaginal microbiota is dominated by *Lactobacillus* spp., often with a single species constituting >50% of the community [1]. These commensal bacteria help exclude pathogens from the vagina and upper reproductive tract by fermenting vaginal glycogen into lactic acid and secreting antimicrobial peptides [2–4]. In bacterial vaginosis (BV), diverse anaerobic and facultative bacteria increase in abundance compared to commensal lactobacilli [5]. These BV-associated bacteria (BVAB) perform functions that can be detrimental to host health, such as degrading the cervicovaginal mucus barrier [6], attacking host cells with cytotoxins [7], and secreting malodorous amines [8, 9].

BV is a highly prevalent condition, affecting ~29% of reproductive-aged women in the USA as assessed by Gram stain of vaginal fluid [10]. Women from disadvantaged socioeconomic groups are at higher risk of BV [11, 12], and having a BV bacterial community is associated with increased risk of adverse sequelae such as pelvic inflammatory disease, preterm birth, and acquisition of sexually transmitted infections [13–16]. Although antibiotic therapy is effective in treating BV, up to 60% of patients experience a recurrence within 1 year [17, 18]. Alternative and supplemental therapies to antibiotics have only modestly improved rates of cure or recurrence [19–21], highlighting the need for a better understanding of BV pathogenesis.

BV is a polymicrobial condition of the vaginal microbiota, and no single organism is sufficient to cause disease [22]. BV is notable for the presence of heterogeneous bacterial communities, further complicating the study of this condition [1]. Interbacterial interactions are thought to be important in maintaining this diverse community structure. Previous *in vitro* studies have found examples of interbacterial relationships, such as *Prevotella bivia* feeding ammonia to *Gardnerella* and *Peptostreptococcus* [23–25], and *Gardnerella vaginalis* promoting the growth of *Fannyhessea vaginae* in a biofilm culture system [26]. Known interactions between BVAB only involve a small number of taxa, however, and other interactions likely exist that help stabilize the BV community. Host–bacterial interactions also likely play a role in BV. Bacterial catabolism of host-derived amino acids into foul-smelling amines such as cadaverine and putrescine is thought to contribute to BV malodor [27]. Similarly, bacterial breakdown of host mucus likely contributes to reduced mucosal barrier capacity and the characteristic thin vaginal discharge [28]. But less is known about the host response to BV and how it can ameliorate or exacerbate the condition.

Untargeted metaproteomics is a powerful tool to study biological processes in complex cellular communities such as those found in the human vagina. By identifying proteins present in the vagina and associating them with the host or specific bacterial taxa, functions that influence the composition of the microbiota can be identified [29]. In this study, we analyze metaproteomic data from cervicovaginal lavage (CVL) samples from 20 women with BV and 9 without BV to uncover *in vivo* host and bacterial processes. We use a high-level, statistical approach to determine what host and bacterial proteins are differentially abundant based on BV status. We also apply a low-level analysis, examining proteins with low spectral counts but biologically important functions. This combination of high- and low-level analysis reveals broad functional differences between the BV and non-BV proteome, which may contribute to the condition, in addition to novel capabilities and interactions in the BV microbiota, which could represent promising targets for disruption by future therapeutics.

## Materials and methods

### Study population and sample collection

The study and sample collection methods were approved by the Institutional Review Board at the Fred Hutchinson Cancer Center and the University of Washington. All study participants provided written informed consent. Participants were recruited from the Seattle & King County Sexual Health Clinic, University of Washington Infectious Diseases/Virology Research Clinic, Hall Health Student Clinic at the University of Washington, University of Washington OB-GYN Clinic, and Harborview Vaginitis Clinic. A random cross-sectional case–control set of 21 samples from women with BV and 9 samples from women without BV were selected for proteomics and metagenomics (Supplementary Table S1). As there is greater bacterial heterogeneity in BV, we used a 2:1 case-to-control ratio for sample collection. One sample could not be analyzed leaving 29 total. CVL samples were collected for molecular characterization, Gram staining, pH, saline microscopy, and potassium hydroxide preparation. CVL was collected by instilling 10 ml sterile saline into the vagina using a needleless syringe, and walls of the vagina were washed to remove any adherent cells. After ~1 min, the lavage fluid was aspirated and stored at −80°C. BV was diagnosed using the Amsel clinical criteria [30] and confirmed by Gram stain using the Nugent and Hillier method [31]. BV diagnosis was concordant by both Nugent score and Amsel criteria for all 29 participants whose samples were analyzed by mass spectrometry. CVL was used for proteomic analyses. A separate set of 60 CVL samples that were used for formic acid quantitation were collected as part of the same parent study, and Nugent score was used to determine their BV status.

CVF samples used for confirmation of transglutaminase three levels were self-collected by participants for a longitudinal study of BV and HSV-2 infection [32]. Participants inserted a menstrual cup (SoftCup) into the vagina for 5–10 min. The menstrual cup was placed in a 50-ml conical tube, kept in the participant’s freezer, and subsequently stored at −80°C until processing. BV status for participants was assessed by Nugent score.

### Swab preparation and DNA extraction from vaginal swab samples

Vaginal swabs collected from study participants were frozen at −80°C until processing. Swabs were prepared for extraction by placing swab tip in a tube with 500 μl filtered (100 K MWCO) 0.9% saline. Swabs were vortexed 1–2 min and then removed. Vaginal fluid from the swab tip was centrifuged at 14 000 rpm for 10 min at 4°C to pellet cells. Genomic DNA (gDNA) was extracted from pellets using the BiOstic Bacteremia DNA Isolation Kit (Qiagen, Germantown, MD, USA). DNA was eluted in 150 μl buffer. DNA extraction controls (blank swab without contact with human mucosa) were included for every 15 samples to assess contamination from extraction reagents or collection swabs. DNA was stored at −80°C until analysis.

### Broad-range polymerase chain reaction (PCR) and sequencing for microbiota characterization

Total bacterial DNA concentrations (16S rRNA gene copies) were measured using a quantitative PCR (qPCR) assay targeting the V3–V4 region of the 16S rRNA gene [1]. Samples were evaluated for the presence of PCR inhibitors using a qPCR assay targeting a segment of exogenously added jellyfish DNA and inhibition was defined as a delay in the threshold cycle (*C*_t_) of >2 cycles compared to no-template controls [33]. Relative abundances of bacterial sequence reads were measured using broad-range PCR targeting the V3–V4 region of the 16S rRNA gene and sequencing on the MiSeq System (Illumina San Diego, CA) [34]. Raw sequence reads were demultiplexed using the MiSeq’s System’s onboard software. Demultiplexed reads were processed using *barcodecop* v0.4.1 (Hoffman N.G., barcodecop, 2019, https://github.com/nhoffman/barcodecop) to enforce barcode quality using default settings and to ensure exact barcode matches to the forward and reverse reads. The *DADA2* package version 1.6.0 was used for quality filtering, read trimming, error correction and dereplication, paired-end assembly, and chimera removal resulting in a list of unique sequence variants [35]. Sequence variants were classified using the phylogenetic placement tool *pplacer* [36] and a curated set of urogenital bacteria [37]. Sequence reads are available from the NCBI Short Read Archive (Accession number PRJNA881379).

### Metagenomics library preparation and sequencing

gDNA from vaginal samples was quantified via Qubit fluorometer and Quant-iT dsDNA Assay Kit, high sensitivity (Life Technologies-Invitrogen, Carlsbad, CA, USA). Sequencing controls included a bacterial mock community (ATCC MSA-1003, Manassas, VA, USA) and a bacterial isolate DNF00720 *F. vaginae*. Sequencing libraries were prepared from 250 pg gDNA with a quarter reaction workflow using the Nextera XT Library Prep Kit (Illumina, San Diego, CA, USA). Libraries were pooled by volume, and post-amplification cleanup was performed using 0.8× Agencourt AMPure XP beads (Beckman Coulter, Indianapolis, IN, USA). The library pool size distribution was validated using the Agilent High Sensitivity D5000 ScreenTape run on an Agilent 4200 TapeStation (Agilent Technologies, Inc., Santa Clara, CA, USA). Additional library QC and cluster optimization were performed using Life Technologies-Invitrogen Qubit 2.0 Fluorometer (Life Technologies-Invitrogen, Carlsbad, CA, USA). Sequencing was performed on a NovaSeq 6000 S1-300 flowcell (Illumina, San Diego, CA, USA). Image analysis and base calling were performed on-board the NovaSeq 6000 using Real Time Analysis v3.4.4 software. Generation of Fastq files was performed with Illumina’s bcl2fastq 2.20 Conversion software. The *geneshot* workflow was used to process Fastq files [38]. This workflow fed data into metaSPAdes for assembly and Prokka for annotation of bacterial genes [39, 40]. Taxonomic annotation was performed using MetaPhlAn2 [41].

### Proteomic sample preparation

Cervicovaginal lavage samples were reduced, denatured, and digested to peptides for mass spectrometry analysis. First, 105 mg of solid urea was added to 250 μl of CVL to yield a final concentration of 7 M. Dithiothreitol was then added to a final concentration of 5 mM, and the sample was incubated for 30 min at 60°C with gentle shaking (300 rpm) in a thermomixer. Following incubation, 2.25 ml of 50 mM ammonium bicarbonate was added to each tube. Trypsin (U.S. Pharmacopeia, Rockville, MD) was resuspended to 1 μg/μl in acetic acid, and 5 μl was added to each sample. The samples were incubated at 37°C with gentle shaking (300 rpm) overnight. Digested peptide samples were centrifuged for 5 min at 12 000 × *g* to pellet any remaining solid debris, and the supernatant was subjected to solid phase extraction (SPE). SPE cartridges (Phenomenex; Strata C18-T (55 μm, 140 Å), 100 mg/1 ml, cat # 8B-S004-EAK) were loaded into the vacuum manifold, and the samples were processed according to the manufacturer’s recommendation. Briefly, cartridges were conditioned using 1 ml methanol and washed using 1 ml 0.1% trifluoroacetic acid (TFA) in water. The sample was added to the cartridge, followed by a wash with 1 ml 5% acetonitrile/95% 0.1% TFA in water. Finally, the sample was eluted with 1 ml 80% acetonitrile and 20% 0.1% TFA in water. The samples were concentrated to near dryness using a SpeedVac and resuspended in 30 μl 0.1% TFA water. A BCA assay (Pierce) was performed to determine peptide concentration. The samples were diluted to 0.1 μg/μl and stored at -80°C until MS analysis.

### Liquid chromatography with mass spectrometry/mass spectrometry analysis

A Waters nano-Acquity dual pumping UPLC system (Milford, MA) was configured for on-line trapping of a 5 μl injection at 5 μl/min with reverse-flow elution onto the analytical column at 300 nl/min. Columns were packed in-house using 360 μm o.d. fused silica (Polymicro Technologies Inc., Phoenix, AZ) with 2 mm sol–gel frits for media retention and contained Jupiter C18 media (Phenomenex, Torrence, CA) in 5 μm particle size for the trapping column (150 μm i.d. × 4 cm long), with 3 μm particle size for the analytical column (75 μm i.d. × 70 cm long). Mobile phases consisted of (A) 0.1% formic acid in water and (B) 0.1% formic acid in acetonitrile with the following gradient profile (min, %B): 0, 1; 2, 8; 20, 12; 75, 30; 97, 45; 100, 95; 110, 95; 115, 1; 150, 1. MS analysis was performed using a Velos Orbitrap mass spectrometer (Thermo Scientific, San Jose, CA) outfitted with a custom electrospray ionization (ESI) interface. Electrospray emitters were custom made by chemically etching 150 μm o.d. × 20 μm i.d. fused silica [42]. The heated capillary temperature and spray voltage were 350°C and 2.3 kV, respectively. Data were acquired for 100 min after a 15-min delay from when the gradient started. Orbitrap spectra (AGC 1 × 106) were collected from 400 to 2000 m/z at a resolution of 60 k followed by data-dependent ion trap MS/MS (collision energy 35%, AGC 1 × 10^4^) of the 10 most abundant ions. A dynamic exclusion time of 45 s was used to discriminate against previously analyzed ions using a −0.55- to 1.55-Da mass window. Each sample was analyzed in two separate replicates.

### Database construction for identification of peptide sequences

A sample-specific database was built to separately analyze each sample, as described previously [43]. Briefly, all databases included human protein sequences from Swiss-Prot (release 2019_02), 16 common contaminants (detailed below), translated bacterial genes identified in the sample by shotgun metagenomic sequencing, and RefSeq protein sequences for bacterial taxa present in the sample at >0.1% abundance according to 16S rRNA gene sequencing (RefSeq release 99, downloaded 19 June 2020) except *Amygdalobacter indicium* and *Amygdalobacter nucleatus* for which there were no available genomes at time of analysis [44]. All bacterial protein sequences were normalized before being incorporated into databases using the SpeciesSeqPrepper.py program. *Gardnerella* species were delineated using the same groups described in Vaneechoutte *et al.* [45]. Recently published *Gardnerella* genomes were submitted to the DSMZ genome-to-genome distance calculator [46], and a cutoff of 70% similarity was used to determine *Gardnerella* species.

### Database searching

Approximately half of bacterial peptides identified in searches of the first replicates were not identified in the second replicates and vice versa, so both replicates for each sample were combined for further proteomic analysis. Peptide identification was performed using MS-GF+ (v2019.01.22). Isotope error range was −1 to 2, maximum modifications per peptide was set to 3, and peptides were only considered if they were at least partially tryptic. The Nextflow workflow manager (v19.07.0) was used to parallelize and automate the data analysis pipeline.

A two-step database search method was used to maximize data from each sample [47]. Briefly, after an initial database search was completed, every protein linked to a peptide-spectrum match (PSM)—regardless of statistical significance—was recorded, and their sequences were included in a subset protein database. A second search was performed using these subset databases so that only proteins identified in the first search were compared against the sample spectra, and the results of these second searches were used for downstream analysis. A decoy database was created and searched concurrently to calculate false discovery rate (FDR) and *Q*-values.

### Proteomic data analysis

Search results were analyzed in JupyterLab (v1.1.4) using Python (v3.6.4). FDR was limited by only including PSMs with a *Q*-value <0.01. To identify sample proteins, peptide spectra were classified in a hierarchical manner. If the spectrum matched any decoy proteins, it was flagged as decoy identification and discarded. Next, if the spectrum matched any contaminant proteins, it was flagged as contaminant identification and similarly discarded. Otherwise, if the spectrum matched any human proteins in the database, it was categorized preferentially as human*.* Finally, the spectrum was characterized as bacterial if it only matched bacterial proteins. Peptides were included in further analysis if MS-GF+ assigned the PSM a spectral probability value <1E-15 or the peptide was one of two unique peptides that matched to the same protein.

### Differential protein abundance analysis

To calculate the relative abundance of human proteins, the total number of PSMs attributed to a protein in a sample was divided by the total number of human PSMs in that sample. Due to their heterogeneity, bacterial proteins were grouped functionally by annotating them with the eggNOG-Mapper web server (v1.0.3) [48, 49]. For bacterial PSMs that matched multiple proteins, only the first protein match was queried. Proteins were then grouped based on the “Preferred_name” field assigned to each by eggNOG-Mapper, and the relative abundance of each group in a sample was determined by dividing the number of PSMs matching that group by the total number of bacterial PSMs in that sample. Protein relative abundances were then log transformed (Base 2) for statistical analysis. Statistical tests were performed using the scipy (v1.3.1) “stats” package. Mann–Whitney *U* tests were used to assess differences in relative abundance of specific proteins across samples.

### Taxonomic analysis of quantitative PCR, DNA sequencing, and proteomic data

Taxonomic analysis was performed at the genus level to better compare data between techniques. For qPCR data, the number of reads for each genus was summed and then divided by the combined number of reads for all qPCR assays performed on the sample to calculate relative abundance. For shotgun metagenomic sequencing data, “estimated_number_of_reads_from_the_clade” value for each genus in the MetaPhlAn2 output was divided by the total number of reads in the sample to calculate relative abundance of each genus. Genera with relative abundance <0.5% were excluded to minimize false-positive identifications. Taxonomic assignment of peptides was performed using the taxonomic information attached to protein sequences in each database. When analyzing taxonomic makeup of samples, spectra that matched multiple genera were excluded, so ambiguous hits would not skew analysis. When analyzing identified bacterial proteins for biological relevance, all taxa-matching sample spectra were noted so functions of minority organisms would not be excluded.

### Contaminant protein sequences

Proteins such as keratins and trypsins are commonly introduced into samples by processing procedures and from the researchers themselves. Therefore, the following contaminant proteins were included in all metaproteomic databases as a minimal set of peptides that should be excluded from downstream analysis while simultaneously attempting to preserve true identifications. These proteins were *Sus scrofa* trypsin precursor (sp|P00761|TRYP_PIG), Promega trypsin artifact 1 (Trypa1), Promega trypsin artifact 2 (Trypa2), Promega trypsin artifact 3 (Trypa3), Promega trypsin artifact 4 (Trypa4), Promega trypsin artifact 5 (Trypa5), Trypsin artifact 6 (Trypa6), *Bos taurus* trypsinogen (sp|P00760|TRYP_BOVIN), *B. taurus* chymotrypsinogen A (CTRA_BOVIN), *B. taurus* chymotrypsinogen B (CTRB_BOVIN), *Homo sapiens* serum albumin precursor (sp|P02768|ALBU_HUMAN), *B. taurus* serum albumin precursor (sp|P02769|ALBU_BOVIN), *H. sapiens* keratin type II cytoskeletal 1 (K2C1_HUMAN), *H. sapiens* keratin type II cytoskeletal 2 (K22E_HUMAN), *H. sapiens* keratin type I cytoskeletal 9 (K1C9_HUMAN), and *H. sapiens* keratin type I cytoskeletal 10 (K1C10_HUMAN).

### Protein homolog searching with BLASTp

The stand-alone Protein–Protein BLAST program (v2.12.0+) was used to identify protein homologs in bacterial genomes. Query sequences were searched against a database that contained all the bacterial proteins used to build the sequence databases for peptide spectrum identification. Default parameters were used for the searches. A bacterial species was determined to possess a homolog of the query protein if at least one isolate encoded a protein that produced a match with an *E*-value <0.0001. The following proteins were used as queries: pyruvate formate-lyase (PFL) sp|P09373|PFLB_ECOLI from *E. coli*, formate dehydrogenase (FDH) sp|P07658|FDHF_ECOLI from *E. coli,* anaerobic ribonucleoside-triphosphate reductase sp|P28903|NRDD_ECOLI from *E. coli*, glutamine synthetase tr|S4HEE6|S4HEE6_GARVA from *G. vaginalis,* glutamate dehydrogenase sp|P00370|DHE4_ECOLI from *E. coli,* glutamate transporter tr|S4HL05|S4HL05_GARVA from *G. vaginalis,* TMAO reductase sp|P33225|TORA_ECOLI from *E. coli*, betaine reductase sp|O69407|GRDH_PEPAC from *Peptoclostridium acidaminophilum,* choline trimethylamine-lyase (CutC) sp|Q30W70|CUTC_OLEA2 from *Oleidesulfovibrio alaskensis,* carnitine monooxygenase (CntA) sp|P0ABR7|CNTA_ECOLI from *E. coli,* arginine deiminase (ADI) sp|P13981|ARCA_PSEAE from *Pseudomonas aeruginosa,* ornithine carbamoyltransferase (OCT) sp|P08308|OTCC_PSEAE from *P. aeruginosa,* ornithine decarboxylase (ODC) sp|P24169|DCOS_ECOLI from *E. coli,* agmatinase sp|P60651|SPEB_ECOLI from *E. coli*, putrescine carbamoyltransferase sp|Q837U7|PTC_ENTFA from *Enterococcus faecalis,* acetylpolyamine amidohydrolase 2 sp|Q9I6H0|APAH2_PSEAE from *P. aeruginosa,* and putrescine-pyruvate aminotransferase sp|Q9I6J2|SPUC_PSEAE from *P. aeruginosa*.

Bacterial proteins identified in the CVL samples were searched for homologs of proteins of interest as described above, using a database populated with all bacterial proteins identified in the samples.

### Screening bacterial isolates for formic acid fermentation, utilization, and depletion

The bacterial isolates used in these experiments were *Lactobacillus crispatus* ATCC33197, *Lactobacillus iners* DSM13335, *Lactobacillus gasseri* DSM20243, *Lactobacillus jensenii* DSM20557, *G. vaginalis* ATCC14018, *G. vaginalis* UPII 315-A, *Gardnerella piotii* JCP8066, *Gardnerella leopoldii* CCUG72425, *Gardnerella swidsinskii* CCUG72429, *F. vaginae* DSM15829, *A. indicium* UPII 610-J, *A. nucleatus* KA00274, *Megasphaera lornae* UPII 199-6, *Megasphaera vaginalis* BV3C16-1, *P. bivia* DSM20514, *Sneathia vaginalis* DSM16631, and *Coriobacteriales* bacterium DNF00809. The lactobacilli, *G. vaginalis*, and *G. piotii* isolates were grown on NYCIII 1.5% agar plates. The *G. leopoldii, G. swidsinskii, F. vaginae, Amygdalobacter, Megasphaera, Prevotella,* and DNF00809 isolates were grown on Brucella H&K agar plates (Hardy Diagnostics, Santa Maria, CA). The *S. vaginalis* isolate was recovered from frozen growth directly in pBHI +5% fetal calf serum (FCS) liquid media. The lactobacilli, *Gardnerella,* and *F. vaginae* isolates were cultured in NYCIII liquid media. The *Megasphaera* and *P. bivia* isolates were cultured in PYG-mod-YG liquid media. The *Amygdalobacter* isolates were cultured in CRM-YG + 3% FCS liquid media. The *Sneathia* and DNF00809 isolates were cultured in WC + 3% FCS liquid media. At each subculture, Gram stains were performed to verify culture purity. All culturing took place in an AS-580 anaerobic chamber (Anaerobe Systems, Morgan Hill, CA) in a gas mixture of 5% CO_2_, 5% H_2_, and 90% N_2_ at 37°C.

To test bacteria for formic acid fermentation, the isolates were recovered from frozen stocks on solid media for 24–72 h depending on the rate of growth of the isolate. When sufficient growth had been achieved, the bacteria were inoculated into 2 ml liquid culture and allowed to grow for another 24 h. Experimental cultures were then prepared by aliquoting 1 ml liquid media into sterile 1.5-ml tubes (Axygen, Union City, CA) and adding 0 , 10, or 100 μl of filter-sterilized 1 M sodium pyruvate (Sigma-Aldrich, St. Louis, MO) so that the total mass of supplemental pyruvate in each tube was 0, 10, or 100 mmol. About 100 μl of bacterial culture was then inoculated into the tubes. Three replicates were made for each isolate in each pyruvate treatment. Uninoculated control tubes were also made for each media type with all three quantities of added pyruvate. The experimental and control tubes were then incubated for 48 h. Following this incubation, the tubes were removed from the anaerobic chamber and centrifuged at 10 000 × *g* for 10 min. Then 900 μl of supernatant was collected and stored at −20°C until the concentration of formic acid could be assayed. Formic acid concentrations were quantified using a formic acid assay kit (Megazyme, Bray, Ireland). About 10 μl of thawed supernatant was tested according to the assay’s microplate procedure in clear-bottom 96-well plates (Corning Inc., Corning, NY) on a BioTek Epoch 2 plate reader (Agilent, Santa Clara, CA). Bacterial culture supernatant formic acid concentrations were calculated against a standard curve and then matched media blanks were subtracted to determine the concentration of formic acid in the supernatant. This concentration was multiplied by the total volume of the culture to determine the total mmol of formate secreted by the bacteria. Welch’s *T*-tests were performed to compare formate in the supernatants of cultures with 10 or 100 mmol of added sodium pyruvate to the 0 mmol controls. Differences were considered significant if *P* < .05.

To test bacteria for the ability to deplete formate from their media, the isolates were recovered on agar media, then subcultured into 2 ml of liquid media, and cultured for 24 h. Experimental cultures were made by mixing 800 μl liquid media, 100 μl filter-sterilized 500 mM sodium formate (Sigma-Aldrich, St. Louis, MO), and 100 μl of bacterial culture in a sterile 1.5-ml tube (QIAGEN, Hilden, Germany). Three replicate tubes were made for each isolate. In addition, a heat-inactivated control was prepared for each isolate by centrifuging 900 μl of bacterial culture at 10 000 × *g* for 10 min to pellet the cells, removing the supernatant and resuspending the cells in 900 μl fresh media to remove any formate they had secreted while growing, and then placing the tubes in a water bath at 70°C for 45 min. The control tubes were allowed to cool and then 100 μl 500 mM sodium formate was added. The control and experimental tubes were incubated for 48 h, removed from the anaerobic chamber, centrifuged at 10 000 × *g* for 10 min, and then 800 μl of supernatant was removed and stored at −20°C until the remaining formate could be assayed. Thawed supernatants were diluted 1:2 and then the concentration of formate was measured using the assay kit as described above. The concentration of formate remaining in the supernatants of the experimental cultures was normalized against their heat-inactivated controls.

To test bacteria for the ability to utilize formate as a carbon and energy source, the isolates were recovered on agar media, subcultured into 2 ml of liquid media, and cultured for 24 h. No-Glucose media stock solutions were made by preparing liquid media without glucose and using 90% the volume of H_2_O. Experimental media was then made by adding filter-sterilized 500 mM glucose, 500 mM sodium formate, or water at 1:10 volume to the No-Glucose media stock solution. About 180 μl of experimental media was aliquoted into wells on a 96-well plate (Corning Inc., Corning, NY). The plate was sealed with Axygen UltraClear Sealing Film (Axygen, Union City, CA) and then transferred to a PLAS Labs Model 830 glove box (PLAS Labs, Lansing, MI), and OD600 was recorded every 30 min 72 h using a BioTek Epoch 2 plate reader (Agilent, Santa Clara, CA) at 37°C in 10% CO_2_ and 90% N_2_.

### Monitoring bacterial growth with supplemental sodium pyruvate

*G. piotii* JCP8066, *G. swidsinskii* CCUG72429, and *A. nucleatus* KA00274 were recovered from frozen stocks on solid media as described above. Culture purity was confirmed by Gram stain, and then the bacteria were inoculated into 2 ml liquid media and cultured anoxically at 37°C for 24 h. To achieve the same concentrations of pyruvate as were used in the above experiments measuring formic acid production, experimental media was prepared by mixing 1 M filter-sterilized sodium pyruvate to the isolate’s preferred media at a volume ratio of 0:1, 1:100, or 1:10. About 200 μl of experimental media was then aliquoted into wells of a 96-well plate (Corning Inc., Corning, NY), and 20 μl of bacterial culture was inoculated into the media. Four replicate wells were prepared for each combination of isolates and media treatments. The plate was sealed with Axygen UltraClear Sealing Film (Axygen, Union City, CA) and then transferred to a PLAS Labs Model 830 glove box (PLAS Labs, Lansing, MI), and OD600 was recorded every 30 min 72 h using a BioTek Epoch 2 plate reader (Agilent, Santa Clara, CA) at 37°C in 10% CO_2_ and 90% N_2_. Bacterial growth was calculated by subtracting the lowest OD600 measurement in the well from the highest the isolate achieved during the 72 h incubation. Relative growth of bacteria in media supplemented with additional formate was calculated by comparing them to a paired well of the same isolate with no added pyruvate.

### Measuring effect of hypophosphite on bacterial growth and formic acid production

Isolates used in these experiments were the same as those tested for formic acid production, with the addition of *L. crispatus* MV-1A-US and *G. vaginalis* ATCC14019. Bacteria were recovered from frozen stocks on agar plates and then subcultured into liquid media as described previously. A stock solution of 5 M sodium hypophosphite (Thermo Fisher Scientific, Waltham, MA) was prepared and filter-sterilized by passing it through a 0.1-μm PVDF syringe filter (EMD Millipore, Darmstadt, Germany). The stock solution was then serially diluted to 250, 100, and 10 mM. About 20 μl of sodium hypophosphite solutions or H_2_O was added to wells of a sterile 96-well polystyrene plate (Corning Inc., Corning, NY). About 160 μl of media was added to the wells and then 20 μl of bacterial culture was inoculated into wells. Three replicate cultures were prepared for each isolate in each hypophosphite condition. The plate was sealed with Axygen UltraClear Sealing Film (Axygen, Union City, CA), then placed in a BioTek Epoch 2 plate reader (Agilent, Santa Clara, CA) inside a Concept anaerobic chamber (Baker, Sanford, ME), and incubated at 37°C for 48 h, shaking for 30 s every 30 min and then measuring the OD600 of each well. ΔOD600 was calculated for each timepoint by subtracting the initial OD600 reading from the reading at the timepoint. Percent growth was calculated by normalizing measurements to the average ΔOD600 for the three cultures at 0 mM hypophosphite. Significant differences were determined using unpaired *T*-test (*P* < .05).

For bacterial isolates where formic acid inhibition was measured, 100 μl of media was sampled immediately after the final OD600 reading and stored at −20°C. The samples were later thawed and formate concentration was quantified using a formic acid assay kit (Megazyme, Bray, Ireland). Significant differences in formic acid concentration of spent media were determined by unpaired *T*-test (*P* < .05).

### DNF00809 testing for formic acid utilization

*Coriobacteriales* bacterium DNF00809 was recovered from frozen stocks on Brucella plates and then subcultured into WC-3% FCS liquid media. Experimental media was made by preparing WC-3% FCS media without any added glucose and then adding filter-sterilized sodium formate (Sigma-Aldrich, St. Louis, MO) solution to a final concentration of 20 mM formate, or water to serve as a vehicle control. About 100 μl of DNF00809 culture was inoculated into 900 μl of 20 mM formate or vehicle control media and mixed well. About 100 μl sterile media was inoculated into 900 μl 20 mM formate media to serve as a negative control for formate depletion. Three replicate cultures were prepared for each condition. About 100 μl of each culture was immediately sampled for formate quantification and stored at −20°C, and then cultures were incubated at 37°C under anoxic conditions. Every 24 h, another 100 μl of each culture was sampled for formic acid quantification and colony forming unit (CFU) counting. CFUs of input and timepoint samples were quantified by serially diluting to 1E1, 1E2, 1E3, and 1E4 and then spotting 5 10 μl drops onto Brucella plates. CFU counting plates were incubated at 37°C for 48 h, and then visible colonies were counted on a single dilution to calculate CFUs/ml at the timepoint. Once the final sample had been collected, all samples were thawed and analyzed for formate concentrations using a formic acid assay kit (Megazyme, Bray, Ireland). Formate concentrations were normalized to the 0 h concentration in the same sample to calculate percent formate concentration. Significant differences in CFUs/ml between 20 mM formate versus vehicle media and percent formate concentration in DNF00809 versus mock-inoculated controls were calculated using unpaired *T*-test (*P* < .05).

DNF00809 consumption of formate produced by bacteria was tested by culturing *G. vaginalis* 315-A in 4 ml PYG-mod-YG + 3% FCS media under anoxic conditions for 48 h. Three replicate cultures were prepared. About 100 μl samples of each culture were taken pre- and post-incubation and stored at −20°C. *G. vaginalis* cultures were then pooled and centrifuged at 10 000 × *g* for 10 min, and then supernatants were filter-sterilized using a 0.1-μm PVDF syringe filter (EMD Millipore, Darmstadt, Germany). About 600 μl spent media were then aliquoted into 1.5-ml tubes (QIAGEN, Hilden, Germany) and 100 μl DNF00809 culture or sterile WC + 3% FCS media were inoculated in. Three replicate tubes were prepared for both experimental and control cultures. The tubes were incubated anoxically at 37°C for 120 h, and then 100 μl were sampled from each. The presence of live DNF00809 bacteria was confirmed by plating 10 μl of each culture onto Brucella plates and allowing them to grow under anoxic conditions at 37°C for 48 h. All samples were thawed, their pH was measured using spotting 30 μl onto MColorpHast pH 4.0–7.0 pH strips (MilliporeSigma, Burlington, MA), and formate concentrations were measured using a formic acid assay kit (Megazyme, Bray, Ireland). Significant differences between DNF00809 cultures and controls were determined using unpaired *T*-test (*P* < .05).

### Measurement of formic acid concentrations in cervicovaginal lavage

Formic acid concentrations were quantified using a formic acid assay kit (Megazyme, Bray, Ireland). CVL samples were thawed and briefly vortexed to homogenize, and then 20 μl of sample and standards were tested according to the assay’s microplate procedure in clear-bottom 96-well plates (Corning Inc., Corning, NY) on a BioTek Epoch 2 plate reader (Agilent, Santa Clara, CA). Three blanks were tested alongside each batch of samples, and the limit of detection for the assay was defined as a blank-adjusted change in OD340 greater than the mean for the three blanks. This equaled a minimum detectable formate concentration of 0.128 mM. Sample formate concentrations were then calculated against a standard curve.

### Quantification of polyamine concentrations in bacterial culture supernatants

The bacterial isolates used in these experiments were *Dialister micraerophilus* DSM19965, *D. micraerophilus* DNF00843, *F. vaginae* DSM15829, *Peptoniphilus lacrimalis* DNF00528, and *G. vaginalis* ATCC14018. The bacteria were first grown in pure culture by recovering them from frozen stocks on Brucella H&K agar plates (Hardy Diagnostics, Santa Maria, CA), followed by two subcultures into 2 ml Brucella H&K media. At each step, bacteria were grown in an AS-580 anaerobic chamber (Anaerobe Systems, Morgan Hill, CA) in a gas mixture of 5% CO_2_, 5% H_2_, and 90% N_2_ at 37°C for 72 h and purity of the cultures was confirmed between each subculture by Gram stain. These pure cultures of actively growing bacteria were then used to inoculate the experimental cultures. For mono-cultures, 200 μl of bacterial culture was inoculated into 1.8 ml Brucella H&K media. For co-cultures, 100 μl of each bacterial isolate was inoculated into 1.8 ml Brucella H&K media. A mono-culture of each isolate was grown, and a co-culture was set up, so both *D. micraerophilus* isolates grew in co-culture with the *F. vaginae, P. lacrimalis*, and *G. vaginalis* isolates. Three replicates of each culture were made in separate tubes. Experimental cultures were grown anoxically at 37°C for 72 h. Following this incubation, the cultures were briefly vortexed to break up biofilms that had formed on the bottom of culture tubes, and then 1 ml of each culture was removed and centrifuged at 10 000 × *g* for 10 min to pellet cells. The supernatant was removed and stored at −20°C until polyamine quantification was performed.

Polyamine quantification was performed with a Fluorometric Total Polyamine Assay Kit (Sigma-Aldrich, St. Louis, MO). To clean up samples prior to analysis, culture supernatants were thawed and 4 μl Sample Clean-Up Mix was added to 200 μl of supernatant. The mixture was incubated at room temperature for 30 min and then transferred to 10 kDa Cut-Off Corning Spin-X UF Concentrators (Corning Inc., Corning, NY). Cleaned-up samples were diluted 1:100, 1:500, and 1:1000 in ultrapure distilled water (Invitrogen, Waltham, MA). These diluted samples were then analyzed according to the kit specifications. Fluorescence of samples was read on a Biotek FLx800 fluorescence microplate reader (Agilent, Santa Clara, CA). Differences in polyamine concentration between samples were calculated by using Welch’s *T*-test to compare. Differences were considered significant if *P* < .05.

### Targeted liquid chromatography with mass spectrometry for quantification of polyamines

In addition to the *D. micraerophilus* cultures described above, additional cultures with exogenous ornithine were grown. l-Ornithine (Sigma-Aldrich, St. Louis, MO) was dissolved in water to a concentration of 500 μg/ml and then filter-sterilized by passing it through a 0.2 μm sterile nylon filter (Thermo Fisher Scientific, Waltham, MA). About 100 μl of pure culture of *D. micraerophilus* DSM19965 or *D. micraerophilus* DNF00843 and 200 μl of 500 μg/ml L-ornithine were then added to 1.7 ml Brucella H&K liquid media, bringing the final concentration of l-ornithine in the culture to 50 μg/ml. Three replicates of these cultures for both *D. micraerophilus* isolates were set up in separate tubes. The bacteria were then cultured anoxically at 37°C for 72 h, and then culture supernatants were collected as described above. Prior to preparation for liquid chromatography with mass spectrometry (LC-MS) analysis, culture supernatants were thawed, and 50 μl of each were pooled, briefly vortexed, and then stored at −20°C until sample preparation was performed.

Four polyamines (putrescine, cadaverine, spermidine, and spermine) for targeted LC-MS analysis were extracted using a protein precipitation method. The samples were first homogenized in 200 μl purified deionized water at 4°C, and then 800 μl of methanol was added. The samples were vortexed, stored for 30 min at −20°C, sonicated in an ice bath for 10 min, and centrifuged for 15 min at 18 000 × *g* and 4°C, and then 600 μl of supernatant was collected from each sample. Finally, recovered supernatants were dried on a SpeedVac (Eppendorf, Enfield, CT) and reconstituted in 0.5 ml of LC-matching solvent for LC-MS acquisition.

Relative quantitation of the four polyamines was performed on a LC-MS system composed of two Shimadzu UPLC pumps (Shimadzu, Kyoto, Japan), CTC Analytics PAL HTC-xt temperature-controlled auto-sampler (CTC Analytics, Zwingen, Switzerland), and AB Sciex 6500+ Triple Quadrupole MS equipped with ESI source (AB SCIEX, Toronto, Canada). The four polyamines were separated on a Waters XBridge BEH Amide column (Waters, Milford, MA) running in hydrophilic interaction liquid chromatography mode. Solvent A consisted of 10 mM ammonium acetate in 95% water, 2% MeOH, 3% acetonitrile, and 0.2% acetic acid. Solvent B consisted of 10 mM ammonium acetate in 5% water, 2% MeOH, 93% acetonitrile, and 0.2% acetic acid. The total chromatography time was 18 min, followed by 18 min for reconditioning the column. The retention times for putrescine, cadaverine, spermidine, and spermine were 9.03, 8.90, 10.28, and 8.51 min, respectively. The separated molecules were ionized in a positive ionization mode (ESI+), and MS data acquisition was performed in multiple reaction monitoring (MRM) mode. The MRM transitions (precursor/product ion pairs) for putrescine, cadaverine, spermidine, and spermine were 89^+^/72^+^, 103^+^/86^+^, 146^+^/72^+^and 203^+^/129^+^, respectively. The LC-MS platform was controlled using AB Sciex Analyst 1.7.2 software. Measured MS peaks were integrated using AB Sciex MultiQuant 3.0.3 software.

### Transglutaminase 3 quantification

10× protease inhibitor solution was made by dissolving two tablets of cOmplete, Mini, ethylenediaminetetraacetic acid (EDTA)-free protease inhibitor cocktail (Roche, Basel, Switzerland) in 1 ml of 100 mM HEPES buffer at pH 8.5 (Sigma-Aldrich, St. Louis, MO). Cervicovaginal fluid (CVF) samples were thawed and briefly vortexed to homogenize them, and then 150 μl of CVF was added to 150 μl of 1× protease inhibitor solution and stored at −80°C. Concentration of transglutaminase 3 in the samples was quantified using a human transglutaminase 3 enzyme-linked immunosorbent assay (ELISA) kit (Invitrogen, Waltham, MA). The samples were serially diluted 1:20, 1:200, 1:1000, 1:2000, and 1:10 000 in 1× assay diluent D, treated according to kit specifications, then the absorbance of the samples was measured at 450 nm, and the concentration was calculated against a standard curve. The scipy (v1.3.1) “stats” package was used to perform a Welch’s *T*-test to compare the average concentration of transglutaminase 3 in CVF samples from participants with BV to those without BV.

## Results

### Bacterial vaginosis is associated with proteins reflecting more diversified bacterial metabolism

We analyzed CVL samples from 20 women with BV (BV+) and 9 women without BV (BV−) by species-specific qPCR, broad-range 16S rRNA gene sequencing, metagenomic sequencing, and liquid chromatography with tandem mass spectrometry (LC-MS/MS) (Supplementary Table S1) [43]. The taxonomic makeup of the samples was generally similar between these four methods (Supplementary Fig. S1). One exception was sample BV+_6, which was dominated by *Candidatus Lachnocurva*, but metagenomic sequencing indicated that this sample mostly contained *Megasphaera*, likely reflecting an absence of *Candidatus Lachnocurva* nucleotide sequences in the metagenomic database. Some genera were over- or under-represented in the metaproteomic data. In samples containing *Gardnerella*, this genus had on average a 2.2× higher abundance in the peptide data than in 16S rRNA gene sequencing data. Conversely, *Sneathia* had a 4.7× lower abundance on average in metaproteomic data than in 16S rRNA gene sequencing. It is unclear whether these differences reflect differential metabolic activity by these taxa, or greater availability of *Gardnerella* protein sequences in our database.

**Figure 1:**
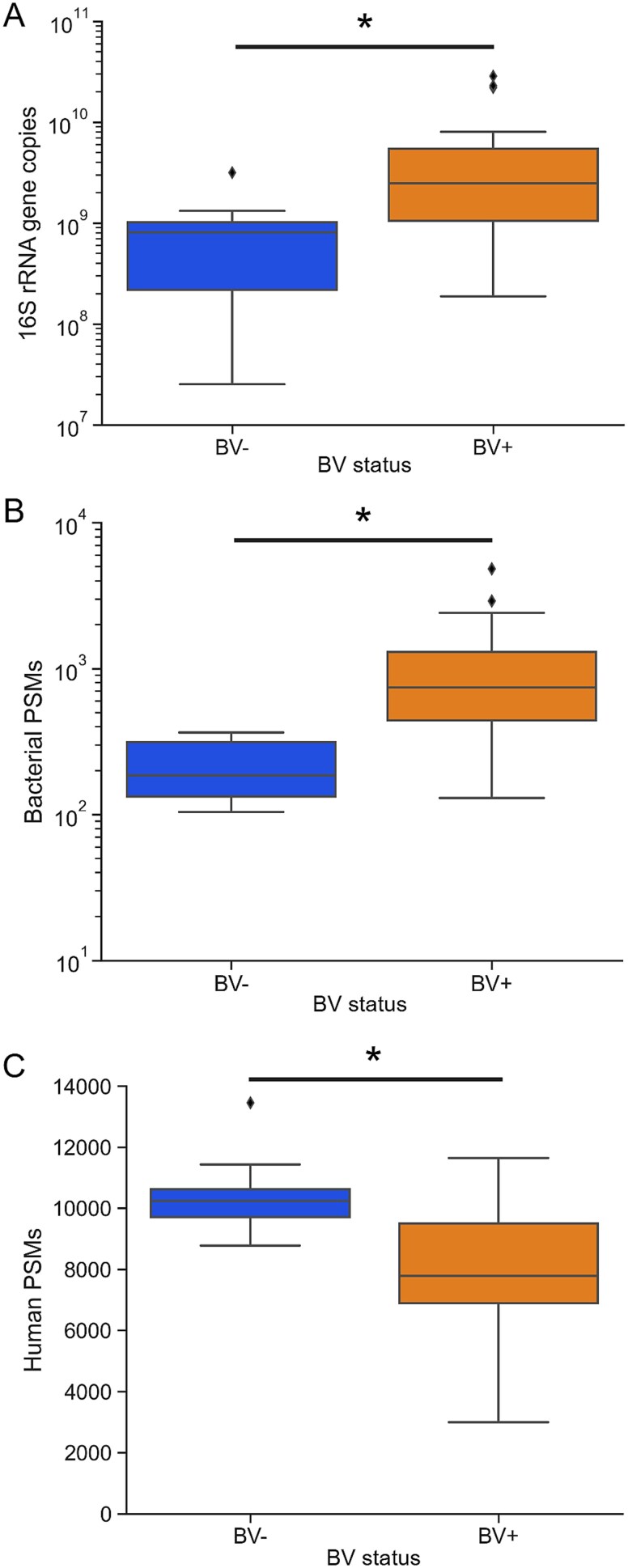
Signs of increased bacterial concentration and metabolism in BV. (A) Total bacterial load as measured by 16S rRNA gene copy number for samples. (B) Number of significant bacterial PSMs identified in each sample. (C) Number of significant human PSMs identified in each sample. Stars show comparisons that were significantly different between BV status by Mann–Whitney *U* test (*P* < .01).

In total, we identified 1068 unique human proteins and 1418 unique bacterial proteins (Supplementary Tables S2 and S3). To elucidate functional differences in the vaginal proteome during BV, we first determined what proteins were significantly differentially abundant based on BV status. To account for a lower number of human PSMs in BV+ samples, we separately normalized the abundance of each human protein to the total number of human spectra identified in each sample, and *vice versa* for bacterial proteins (Supplementary Fig. S2). Bacterial proteins involved in general metabolism such as RNA polymerase, ATP synthase, and ribosomal proteins had significantly higher relative abundances in samples from BV+ participants (Table 1). Bacterial load as measured by total 16S rRNA gene copies per sample was also significantly higher in BV+ samples (Fig. 1A). On average, we identified 5× as many bacterial PSMs in the BV+ samples compared to BV− (Mann–Whitney *U* test, *P* < .001) (Fig. 1B) We also identified significantly fewer human PSMs in BV+ samples (Mann–Whitney *U* test, *P* < .001) (Fig. 1C). Thus, bacterial proteins were a larger share of the overall proteome in BV+ samples.

**Table 1:** Significantly differentially abundant proteins by BV status.

**Protein**	**Host or bacterial**	**Higher/lower abundance in BV**	**Function category**
Fructose-bisphosphate aldolase	Bacterial	Lower	Glycolysis
Pyruvate kinase	Bacterial	Lower	Glycolysis
Enolase	Bacterial	Lower	Glycolysis
Phosphoglycerate kinase	Bacterial	Lower	Glycolysis
6-Phosphofructokinase	Bacterial	Lower	Glycolysis
Glyceraldehyde-3-phosphate dehydrogenase	Bacterial	Lower	Glycolysis
Phosphoglycerate mutase	Bacterial	Lower	Glycolysis
Phosphoenolpyruvate carboxykinase	Bacterial	Higher	Gluconeogenesis
Pyruvate, phosphate dikinase	Bacterial	Higher	Gluconeogenesis
Lactic acid dehydrogenase	Bacterial	Lower	Lactate fermentation
Pyruvate formate-lyase	Bacterial	Higher	Formate fermentation
Alcohol dehydrogenase	Bacterial	Higher	Alcohol fermentation
Acetate kinase	Bacterial	Higher	Acetate fermentation
Extracellular glycosidase	Bacterial	Higher	Polysaccharide breakdown
Glutamate dehydrogenase	Bacterial	Higher	Nitrogen metabolism
Glutamate transporter	Bacterial	Higher	Nitrogen metabolism
Glycine reductase	Bacterial	Higher	Amino acid metabolism
Glutamine synthase	Bacterial	Higher	Amino acid metabolism
Ornithine transcarbamoylase	Bacterial	Higher	Amino acid metabolism
Extracellular peptidase	Bacterial	Higher	Protein breakdown
RNA polymerase	Bacterial	Higher	General metabolism
ATP synthase	Bacterial	Higher	General metabolism
ABC transporter	Bacterial	Higher	General metabolism
Ribosome	Bacterial	Higher	General metabolism
Peroxiredoxin	Bacterial	Higher	Antioxidant
Ladinin-1	Host	Lower	Epithelial structure
Catenin alpha-2	Host	Lower	Epithelial structure
Involucrin	Host	Lower	Epithelial structure
SPR3	Host	Lower	Epithelial structure
Cornifelin	Host	Lower	Epithelial structure
Protein S100-A10	Host	Lower	Epithelial structure
Tight junction protein ZO-1	Host	Lower	Epithelial structure
Cornulin	Host	Lower	Epithelial structure
Periplakin	Host	Lower	Epithelial structure
Zyxin	Host	Lower	Epithelial structure
Filaggrin	Host	Lower	Epithelial structure
Cadherin-1	Host	Higher	Epithelial structure
Cornifin-A	Host	Lower	Epithelial structure
S100-A11	Host	Lower	Epithelial structure
Transglutaminase 3	Host	Higher	Epithelial repair
Pyruvate kinase PKLR	Host	Lower	Glycolysis
Glyceraldehyde-3-phosphate dehydrogenase	Host	Lower	Glycolysis
Phosphoglycerate kinase 2	Host	Lower	Glycolysis
Alpha-enolase	Host	Lower	Glycolysis
ATP-dependent 6-phosphofructokinase, platelet type	Host	Lower	Glycolysis
Glucose-6-phosphate isomerase	Host	Lower	Glycolysis
ATP-dependent 6-phosphofructokinase, liver type	Host	Lower	Glycolysis
l-lactate dehydrogenase A chain	Host	Lower	Acidification
Glycogen phosphorylase, liver form	Host	Lower	Glycogen metabolism
Leukocyte elastase inhibitor	Host	Lower	Protease inhibitor
Calpastatin	Host	Lower	Protease inhibitor
Cystatin-A	Host	Lower	Protease inhibitor
Serpin B4	Host	Lower	Protease inhibitor
Serpin B5	Host	Lower	Protease inhibitor
Cystatin-B	Host	Lower	Protease inhibitor
Serpin B3	Host	Lower	Protease inhibitor
Serpin I2	Host	Lower	Protease inhibitor
Hemopexin	Host	Higher	Heme sequestration
Heme oxygenase 1	Host	Lower	Heme breakdown

Significantly differentially abundant proteins as determined by Mann–Whitney *U* test (*P* < .05) from the host or bacteria. Homologous proteins from different bacterial species were identified and grouped using eggnog-mapper.

Glycolysis enzymes were some of the most identified proteins in our data, but both human and bacterial glycolysis enzymes were less abundant in BV+ samples (Table 1). Conversely, bacterial glycosidases—which can release metabolizable sugars from vaginal glycogen [50]—were more abundant in BV+ samples (Table 1). These results indicate that—although there is increased competition for carbohydrates in BV—carbohydrates make up a smaller overall percentage of bacterial metabolism in BV. This interpretation is supported by the higher abundance of bacterial proteases and amino acid catabolic enzymes in BV+ samples (Table 1).

### *Dialister micraerophilus* cooperates with other bacterial vaginosis-associated bacteria to increase polyamine synthesis

**Figure 2:**
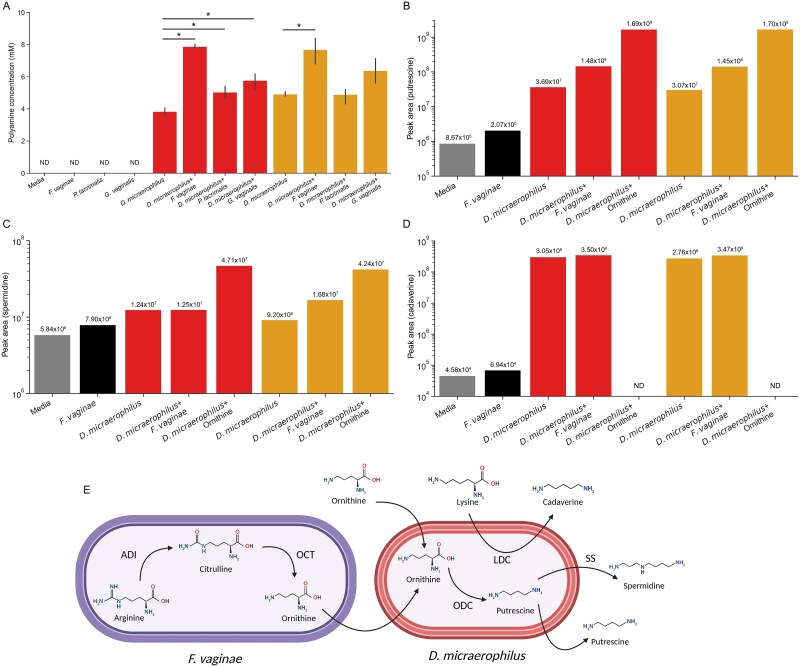
Biosynthesis of polyamines by *D. micraerophilus* and other BVAB. *D. micraerophilus* strains DSM19965 and DNF00843 were grown anaerobically in mono- and co-culture with *F. vaginae* DSM15829, *P. lacrimalis* DNF00528, and *G. vaginalis* ATCC14018 in Brucella H&K media for 72 h and then pelleted, and culture supernatants were collected. Cultures with *D. micraerophilus* DSM19965 are shown in red on left and cultures with *D. micraerophilus* DNF00843 are shown in orange on right. (A) Total polyamine concentration of culture supernatants as measured by enzymatic assay. Bars show standard error of measurements from three separate cultures. ND: Measured polyamine concentrations were below the limit of detection for the assay. Stars show statistically significant differences (Welch’s *T*-test, *P* < .05). Supernatants from three replicates of the *D. micraerophilus* mono-cultures and co-cultures with *F. vaginae*—in addition to cultures supplemented with 50 μg/ml l-ornithine—were pooled and analyzed using LC-MS/MS for (B) putrescine, (C) spermidine, and (D) cadaverine. Numbers above bars show measured peak area for the pooled samples. Spermine was not detected in any culture supernatants. (E) Model of polyamine synthesis by *D. micraerophilus* aided by *F. vaginae*. ADI, arginine deiminase. OCT, ornithine carbamoyltransferase. ODC, ornithine decarboxylase. SS, spermidine synthase. LDC, lysine decarboxylase. Created in BioRender https://BioRender.com/12doaxh.

The concentrations of unpleasant-smelling compounds such as trimethylamine (TMA), cadaverine, and putrescine are commonly elevated in BV, which likely contributes to the malodor associated with the condition [9, 51]. Among the bacterial proteins in our data, we identified a complete enzymatic pathway to convert arginine into putrescine: ADI, OCT, and ODC [52], but different enzymes were associated with different species. We identified spectra-matching ADI and OCT from both *F. vaginae* and *S. vaginalis* (Table 2). Spectra-matching ODC were associated exclusively with *D. micraerophilus*. Genomic analysis supported these proteomic data; *F. vaginae* and *S. vaginalis* genomes lacked ODC, whereas *D. micraerophilus* possessed homologs of ODC but not ADI or OCT (Table 3). None of these organisms possessed homologs of other putrescine-synthesizing enzymes including agmatinase, putrescine carbamoyltransferase, acetylpolyamine amidyhydrolase 2, or putrescine-pyruvate aminotransferase [53–56]. Based on these data, we hypothesized that *F. vaginae* and *D. micraerophilus* can form a syntrophic metabolic relationship to synthesize putrescine. To test this hypothesis, we grew two isolates of *D. micraerophilus* in mono-culture and in co-culture with *F. vaginae* and then quantified the total polyamines present in their culture supernatants using an enzymatic assay. As *G. vaginalis* does not encode putative ornithine-producing enzymes, whereas *P. lacrimalis* encodes a homolog of the ornithine-producing enzyme arginase (Table 3) [57], we also tested mono- and co-cultures with these organisms. *F. vaginae, G. vaginalis,* and *P. lacrimalis* did not synthesize detectable concentrations of polyamines in mono-culture, but both isolates of *D. micraerophilus* produced high concentrations of polyamines (Fig. 2A). In line with our hypothesis, both *D. micraerophilus* isolates produced significantly higher concentrations of polyamines in co-culture with *F. vaginae* (Welch’s *T*-test, *P* < .05). Although *D. micraerophilus* DSM19965 produced a significantly higher concentration of polyamines in co-culture with both *P. lacrimalis* and *G. vaginalis* as expected; differences in polyamine concentrations for the *D. micraerophilus* DNF00843 isolate in co-cultures with these other bacteria were not statistically significant, suggesting strain-level differences in bacterial metabolism.

**Table 2:** Biologically relevant bacterial proteins identified in CVL samples.

**Protein**	**Associated taxa**	**BV− total spectral count**	**BV+ total spectral count**
Type I pullulanase	*Gardnerella*	0	32
Secreted alpha-amylase	*Gardnerella*	0	40
Type I pullulanase	*Lactobacillus crispatus*	6	0
Pyruvate formate-lyase	16 BVAB Genera^a^	0	180
Pyruvate formate-lyase	*Amygdalobacter* (BVAB2)	0	63
Pyruvate formate-lyase	*Megasphaera*	0	52
Pyruvate formate-lyase	*Gardnerella*	0	43
Anaerobic ribonucleoside-triphosphate reductase	*Gardnerella*	31	119
Lactate dehydrogenase	*Lactobacillus*	168	37
Lactate dehydrogenase	*Gardnerella*	0	49
Lactate dehydrogenase	*Candidatus Lachnocurva* (BVAB1)	0	8
Phosphate acetyltransferase	*Candidatus Lachnocurva* (BVAB1)	0	5
Phosphate acetyltransferase	*Gardnerella*	0	52
Acetate kinase	*Gardnerella*	0	32
Acetate kinase	*Gardnerella, Mobiluncus*	1	41
Acetyl-CoA hydrolase	*Megasphaera, Porphyromonas*	0	14
Phosphoenolpyruvate carboxykinase	*Porphyromonas, Prevotella*	0	190
Malate dehydrogenase	*Prevotella*	0	41
Fumarate hydratase	*Prevotella*	0	4
Succinate dehydrogenase	*Porphyromonas, Prevotella*	0	14
Alcohol dehydrogenase	*Gardnerella*	0	72
Alcohol dehydrogenase	Candidatus *Lachnocurva* (BVAB1), *Gardnerella, Mageeibacillus, Parvimonas, Sneathia, Tissierellia*	3	68
Pyruvate:ferredoxin oxidoreductase	*Dialister, Megasphaera, Murdochiella, Peptoniphilus, Porphyromonas, Prevotella, Veillonella*	0	80
Glutamate dehydrogenase	*Aerococcus, Arcanobacterium, Fannyhessea, Gemella, Megasphaera, Mobiluncus, Porphyromonas, Prevotella, Streptococcus*	0	167
Glutamate transporter	*Gardnerella*	0	32
Glutamine synthetase	*Gardnerella*	0	33
Betaine reductase	*Finegoldia, Parvimonas*	0	29
Arginine deiminase	*Fannyhessea vaginae*	0	6
Arginine deiminase	*Sneathia vaginalis*	0	8
Ornithine carbamoyltransferase	*Fannyhessea, Sneathia, Mageeibacillus, Parvimonas*	0	11
Ornithine decarboxylase	*Dialister micraerophilus*	0	9

Bacterial proteins with potential biological significance identified in CVL samples. All taxa associated with proteins matching the sample spectra are listed, along with total spectral count across all BV− or BV+ samples.

a The 16 genera of BVAB associated with pyruvate formate-lyase were *Amygdalobacter* (BVAB2), *Anaerococcus, Arcanobacterium, Atopobium, Bulleidia,* Candidatus *Lachnocurva* (BVAB1), *Fannyhessea, Gardnerella, Gemella, Megasphaera, Peptoniphilus, Porphyromonas, Prevotella, Sneathia, Sutterella,* and *Veillonella*. Spectra that exclusively matched proteins from a single genus are listed on separate rows.

To determine the precise polyamines *D. micraerophilus* synthesized in mono- and co-cultures with *F. vaginae*, we performed targeted metabolomics by liquid chromatography with mass spectrometry (LC-MS) for putrescine, spermidine, spermine, and cadaverine. We pooled supernatants from all three replicates of the *D. micraerophilus* mono- and co-cultures with *F. vaginae* for analysis. We also analyzed cultures of both *D. micraerophilus* isolates in media supplemented with 50 μg/ml L-ornithine to determine whether this precursor alone could increase putrescine biosynthesis. We did not detect spermine in any of the supernatants, but we did find putrescine in all eight pooled culture supernatants (Fig. 2B, Supplementary Table S4). Both *D. micraerophilus* strains made putrescine in mono-culture, possibly because the blood in their rich media supplied some ornithine [58]. The concentration of putrescine was four times higher in co-cultures of *D. micraerophilus* with *F. vaginae* than mono-cultures and much higher when the culture media was supplemented with ornithine, consistent with our hypothesis. Spermidine only increased when exogenous ornithine was added to the culture media (Fig. 2C). Although both *D. micraerophilus* isolates synthesized cadaverine in mono- and co-culture with *F. vaginae*, cadaverine was undetectable in cultures supplemented with ornithine (Fig. 2D). These results suggest that on its own, *D. micraerophilus* can scavenge lysine as a precursor for cadaverine biosynthesis and can make putrescine from ornithine when the latter compound is present in its environment. Other BVAB such as *F. vaginae* might supply additional ornithine to increase putrescine biosynthesis (Fig. 2E). Biosynthesis of cadaverine and putrescine is likely co-regulated in *D. micraerophilus* because an excess of the putrescine precursor ornithine led to the abrogation of cadaverine production.

### Identification of carbohydrate metabolic proteins reveals widespread formic acid fermentation among bacterial vaginosis-associated bacteria and formate utilization by *Coriobacteriales* bacterium DNF00809

**Figure 3:**
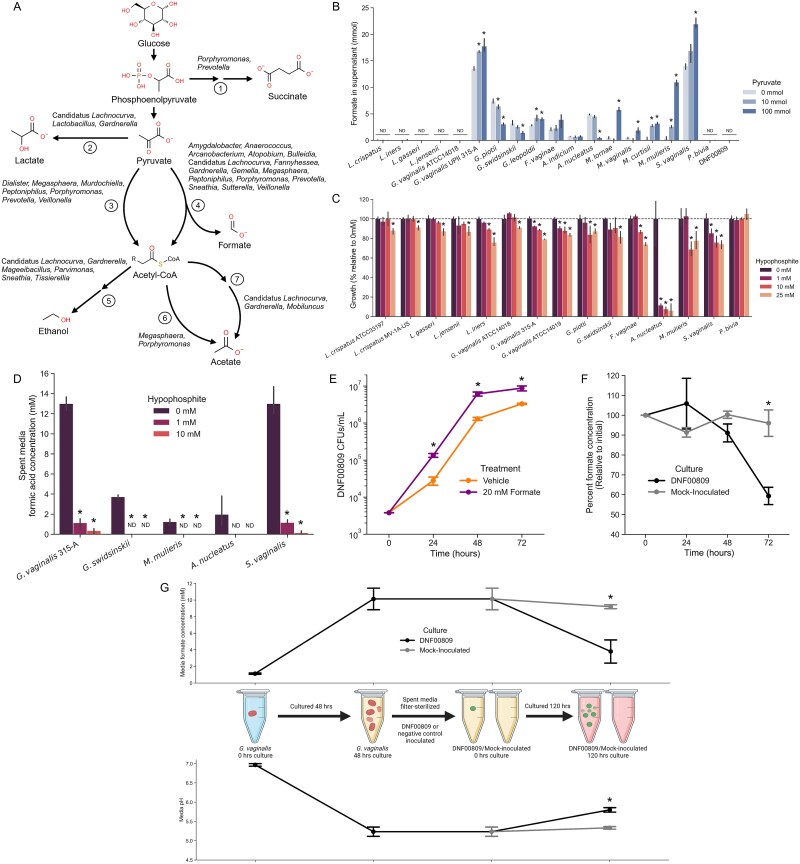
Bacterial fermentation elucidated by metaproteomics. (A) Summary of bacterial fermentation pathways observed in CVL samples. Genera associated with observed sample proteins are listed next to each enzymatic step. Numbers group different enzymatic pathways: (1) phosphoenolpyruvate carboxykinase, malate dehydrogenase, fumarate hydratase, and succinate dehydrogenase. (2) Lactate dehydrogenase. (3) Pyruvate:ferredoxin oxidoreductase. (4) Pyruvate formate-lyase. (5) Acetaldehyde dehydrogenase and alcohol dehydrogenase. (6) Acetyl-CoA hydrolase. (7) Phosphate acetyltransferase and acetate kinase. (B) Total mass of formate present in the supernatant of bacterial cultures grown anaerobically over 72 h. Culture media was supplemented with 0, 10, or 100 mmol of sodium pyruvate at the start of the incubation. Stars indicate the mass of formate was significantly different compared to the 0 mmol control for the same isolate (Welch’s *T*-test, *P* < .05). ND: Formic acid concentration of all replicates was below limit of detection for the assay. Bars show standard error of three replicates. (C) Growth inhibition by the PFL-inhibitor sodium hypophosphite. Bacteria were cultured for 48 h in media with varying concentrations of hypophosphite, and their growth in each condition was calculated by ΔOD600 and then normalized to growth with 0 mM hypophosphite. Dotted line shows 100% growth relative to control. Stars indicate that the growth was significantly lower than control (unpaired *T*-test, *P* < .05). (D) Inhibition of formic acid secretion by hypophosphite. Immediately following hypophosphite growth experiments, spent media of select formate-producing bacteria was sampled and formate concentration was quantified. (E) *Coriobacteriales* bacterium DNF00809 was cultured in media containing 20 mM sodium formate or vehicle. Bacterial density was measured using CFU counting of the inoculum and samples taken every 24 h. Stars indicate a significant difference between treatments (unpaired *T*-test, *P* < .05). (F) Formate was quantified using enzymatic assay in the 20 mM formate DNF00809 culture and a mock-inoculated control. Formate concentration at subsequent time points was then normalized to concentration of the culture at the beginning of the experiment. Star indicates significant difference in percent formate concentration between cultures (unpaired *T*-test *P* < .05). (G) DNF00809 consumption of formic acid produced by *G. vaginalis*. *G. vaginalis* 315-A was cultured for 48 h and samples of media were taken pre- and post-incubation to quantify formate concentration and pH. *G. vaginalis* spent media was then filter-sterilized and inoculated with DNF00809 or mock-inoculated, re-cultured for 120 h, and then again sampled for formate concentration and pH. Bars show standard error of three separate cultures. Stars show significant differences between DNF00809 cultures and mock-inoculated controls (unpaired *T*-test, *P* < .05). Created in BioRender https://BioRender.com/fja7pzy.

To determine the functions performed by different organisms *in vivo*, we examined the taxa associated with biologically relevant proteins identified in our metaproteomic data. Glycogen is an abundant source of carbon and energy for vaginal bacteria, but it must be broken down into smaller sugars for cells to import and metabolize [50]. Most glycogen-degrading amylase and pullulanase enzymes exclusively matched *Gardnerella* proteins, but six such spectra from BV− samples matched proteins from *L. crispatus* (Table 2), indicating that these organisms are primarily responsible for glycogen breakdown *in vivo*. Many sample spectra matched bacterial fermentative enzymes, revealing which taxa consumed carbohydrates released from vaginal glycogen and what metabolic end-products are likely produced (Fig. 3A and Table 2). Some spectra matched succinate dehydrogenases from *Porphyromonas* or *Prevotella*, providing evidence these organisms contribute to increased succinate concentrations associated with BV [51]. Acetate-producing enzymes matched proteins from bacteria including *Gardnerella, Megasphaera, Mobiluncus, Porphyromonas,* and *Candidatus Lachnocurva* (alternatively known as BVAB1). Several identified proteins involved in ethanol synthesis were associated with six genera of BVAB (Fig. 3A and Table 2).

The most common bacterial fermentative enzyme in our data was PFL (Table 2), which converts pyruvate into formic acid and acetyl-CoA [59]. Many of the spectra associated with this enzyme exclusively matched proteins from a single bacterial genus including 63 unique to *Amygdalobacter,* 52 unique to *Megasphaera*, and 43 unique to *Gardnerella*. Other PFL spectra matched proteins from many organisms, encompassing 16 different bacterial genera*.* Genomic analysis showed that although these enzymes are absent from commensal lactobacilli, most major BVAB encode putative PFL enzymes (Table 3). Exceptions included the *Prevotella* species *Prevotella amnii, P. bivia,* and *Prevotella timonensis,* which did not have homologs of these proteins.

**Table 3:** Distribution of key metabolic enzymes in vaginal bacteria.

	**Formate**			**Glutamate**			**TMA**		**Putrescine**		
	PFL	FDH	NrdD	GlnA	GdhA	GluA	TorA	GrdH	ADI	OCT	ODC
*Lactobacillus crispatus*			✓	✓		✓			✓		✓
*Lactobacillus iners*			✓	✓		✓					
*Lactobacillus gasseri*			✓	✓		✓					✓
*Lactobacillus jensenii*			✓	✓		✓			✓	✓	✓
*Amygdalobacter indicium* (BVAB2)	✓		✓	✓				✓	✓	✓	
*Amygdalobacter nucleatus*	✓		✓	✓				✓	✓	✓	
Candidatus *Lachnocurva* (BVAB1)	✓		✓		✓	✓			✓	✓	
*Coriobacteriales* bacterium DNF00809		✓	✓	✓	✓		✓			✓	
*Dialister micraerophilus*			✓	✓		✓					✓
*Fannyhessea vaginae*	✓		✓	✓	✓	✓			✓	✓	
*Finegoldia magna*	✓	✓	✓		✓		✓	✓	✓	✓	
*Gardnerella*	✓		✓	✓		✓					
*Mageeibacillus indolicus*	✓		✓			✓			✓	✓	
*Megasphaera hutchinsoni*	✓		✓	✓	✓	✓					
*Megasphaera lornae*	✓	✓	✓	✓	✓	✓	✓	✓		✓	✓
*Mobiluncus curtisii*	✓	✓		✓	✓		✓		✓		
*Mobiluncus mulieris*	✓	✓		✓	✓					✓	
*Parvimonas micra*	✓	✓			✓			✓	✓	✓	
*Peptoniphilus*	✓		✓	✓	✓	✓			✓		
*Porphyromonas*	✓		✓	✓	✓						
*Prevotella amnii*				✓	✓						
*Prevotella bivia*				✓	✓						
*Prevotella timonensis*			✓	✓	✓						
*Sneathia vaginalis*	✓		✓						✓	✓	
TM7		✓	✓	✓	✓					✓	

Presence of a homologous protein for a given bacterial species is represented by a check mark. The cell is empty for species with no significant matches to the query protein. BLASTp searches for reference proteins were performed against all bacterial proteins used to build the sequence databases for this study. A protein was determined to be present in a bacterial species if at least one isolate encoded a homolog for the query sequence with an *E*-value <0.0001. Proteins are grouped by the metabolite associated with their enzymatic activity. Proteins are PFL, pyruvate formate-lyase. FDH, formate dehydrogenase. NrdD, anaerobic ribonucleoside-triphosphate reductase. GlnA, glutamine synthetase. GdhA, glutamate dehydrogenase. GluA, glutamate transporter. TorA, trimethylamine *N*-oxide reductase. GrdH, betaine reductase. ADI, arginine deiminase. OCT, ornithine carbamoyltransferase. ODC, ornithine decarboxylase.

To test whether or not diverse BVAB can produce formic acid, we cultured a selection of vaginal bacteria in media with varying quantities of added pyruvate and then measured formate present in their culture supernatants. In total, 12 species of BVAB secreted detectable quantities of formic acid (Fig. 3B). Most formate-producing bacteria secreted higher concentrations when grown with supplemented pyruvate, except for *G. piotii, G. swidsinskii,* and *A. nucleatus* where increased pyruvate caused these isolates to grow to a lower density (Supplementary Fig. S3A). Bacteria without a PFL enzyme including four species of commensal lactobacilli, *P. bivia,* and *Coriobacteriales* bacterium DNF00809 did not produce measurable quantities of formate (Fig. 3B). The type strain *G. vaginalis* ATCC 14018 is an outlier among this species because its PFL gene contains a premature stop codon, which is likely the reason why we did not detect formate in its spent culture media (Fig. 3B).

Hypophosphite is an antimicrobial and known inhibitor of PFL [60, 61]. We therefore hypothesized that formic acid-secreting bacteria would be more sensitive to hypophosphite. In line with our hypothesis, hypophosphite concentrations as low as 1 mM significantly reduced the growth of formate-producing organisms including *G. vaginalis* 315-A*, A. nucleatus, Mobiluncus mulieris,* and *S. vaginalis* (Fig. 3C and Supplementary Fig. S3B). The effect on *G. piotii, G. swidsinskii,* and *F. vaginae* was less pronounced but trended toward reduced growth. In contrast, hypophosphite had a much smaller effect on growth of organisms that did not secrete formic acid including lactobacilli, *P. bivia*, and *G. vaginalis* ATCC 14018. Hypophosphite concentrations up to 10 mM did not significantly alter growth of *L. crispatus, L. gasseri,* or *L. jensenii*, but *L. iners* was slightly more sensitive (Fig. 3C). We quantified formate in spent media from select organisms grown with hypophosphite and found that although 1 mM hypophosphite only reduced growth by 10%–20%, this concentration nearly abolished formate secretion (Fig. 3D). *G. vaginalis, G. swidsinskii, M. mulieris, Atopobium vaginae,* and *S. vaginalis* all produced approximately 90% lower formic acid concentrations in the presence of 1 mM hypophosphite, with stronger effects at 10 mM. These data support hypophosphite as an efficient inhibitor of bacterial formic acid synthesis and suggest it has stronger antimicrobial properties toward organisms that rely on formate production for their growth.

We next quantified formate in CVL samples from a different cohort of study participants (30 BV+ and 30 BV−) to determine whether or not there was evidence of formic acid fermentation *in vivo*. Of these 60 samples, only 4 had formate concentrations above the assay’s limit of detection (Supplementary Table S5), suggesting that if bacteria secrete formic acid in BV, it does not accumulate to high concentrations. We tested whether isolates from seven vaginal genera could grow on formate or deplete the compound from their media, but did not find evidence of formate consumption or increased growth on formate compared to controls (Supplementary Fig. S3C and D). However, a small number of taxa including *Coriobacteriales* bacterium DNF00809 encode formate dehydrogenase enzymes (FDHs), which may allow them to use formate as a substrate for ATP generation (Table 3) [62]. This fastidious organism has not yet been systematically named, but it is most closely related to *Eggerthella* and is commonly called *Eggerthella*-like vaginal bacterium [63]. Past studies have found this organism in the vaginal communities of 91% of individuals with BV [5], and it was present at up to 6% abundance in our samples (Supplementary Fig. S1). When we cultured DNF00809 with formate, it grew significantly more compared to control media (unpaired *T*-test, *P* < .05) (Fig. 3E). In the same culture, this organism depleted approximately 40% of formate from its media (Fig. 3F). *Coriobacteriales* bacterium DNF00809 depleted a similar percentage of formic acid from *G. vaginalis* spent media and significantly increased the spent media’s pH (unpaired *T*-test, *P* < .05) (Fig. 3G). Therefore, bacteria with FDHs such as *Coriobacteriales* bacterium DNF00809 may serve an important role in the BV microbiota by removing formic acid secreted by other organisms.

### Bacterial nitrogen metabolism revealed by metaproteomics

Metabolism of nitrogen-containing compounds such as proteins and amino acids is thought to be important for BVAB [64]. In our data, we identified extracellular proteases from both *Gardnerella* and *Prevotella* that may contribute to host tissue proteolysis (Table 2). Many spectra matched glutamate dehydrogenases from diverse BVAB, though none matched *Gardnerella* proteins and BLASTp searches of *Gardnerella* genomes failed to identify homologs of this enzyme (Table 3). However, we did find spectra that exclusively matched glutamate transporters and glutamine synthetases from *Gardnerella,* which may allow these bacteria to uptake and incorporate glutamate made by other BVAB [65].

Trimethylamine is a volatile, foul-smelling amine that is present at higher concentrations in women with BV compared to those with an optimal vaginal microbiota [9]. In our data, we identified 29 spectra matching the TMA-producing enzyme betaine reductase (GrdH) from *Parvimonas micra* and *Finegoldia magna* (Table 2) [66]. To determine what other organisms may produce TMA, we searched the genomes of other vaginal bacteria for GrdH in addition to three other TMA-producing enzymes: trimethylamine *N*-oxide reductase (TorA) [67], CutC [68], and CntA [69]. These enzymes were not widely distributed among vaginal bacteria, and neither CutC nor CntA homologs were present in any of the genomes searched (Table 3). GrdH was the most common, with homologs present in *A. indicium* (previously referred to as BVAB2)*, F. magna, P. micra,* and *M. lornae*. Trimethylamine *N*-oxide reductase homologs were also present in *F. magna* and *M. lornae*, in addition to *Mobiluncus curtisii* and *Coriobacteriales* bacterium DNF00809. These results suggest that TMA is likely produced from betaine *in vivo*, though members of the BV microbiota also have the capacity to synthesize this compound from trimethylamine *N*-oxide.

### Signals of host responses to bacterial vaginosis in metaproteomic data

**Figure 4:**
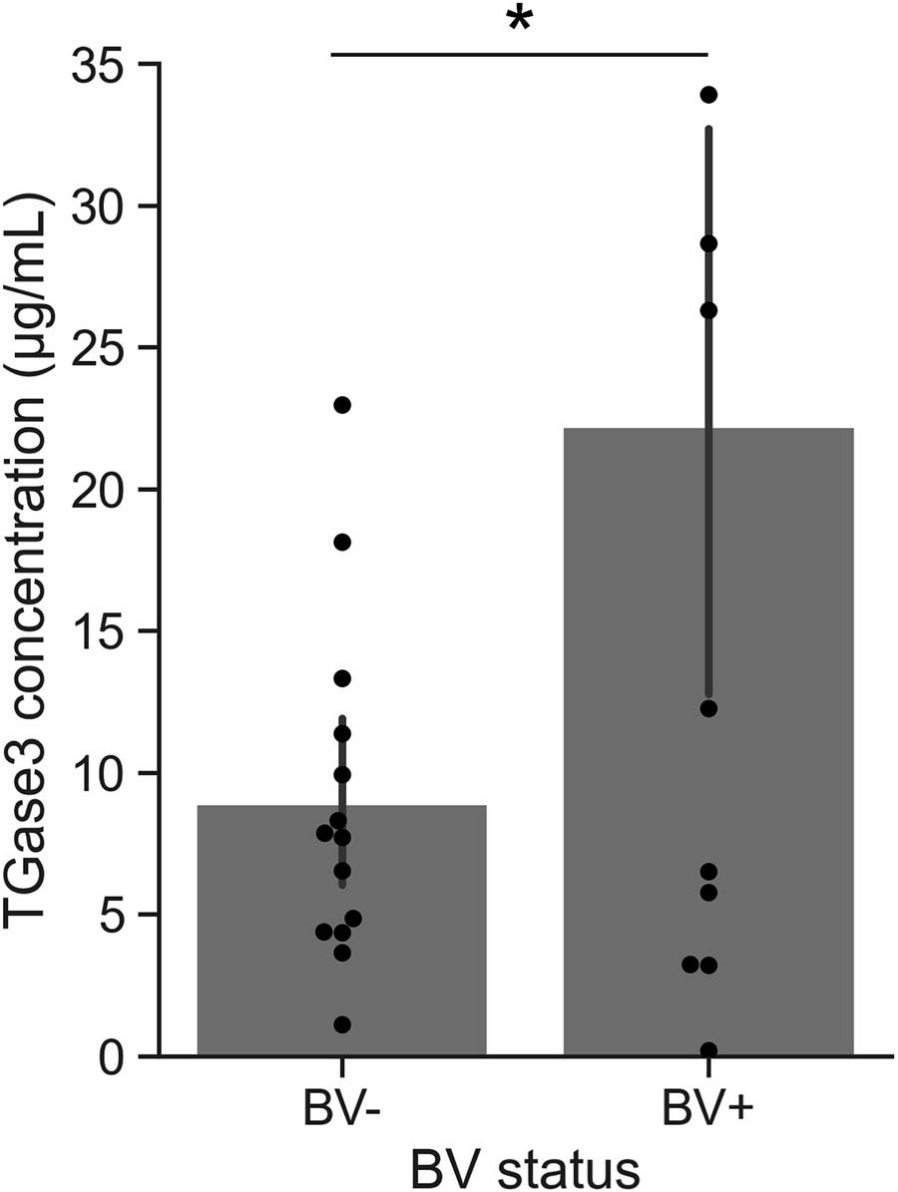
Concentration of human TGase3 in CVF samples as measured by sandwich ELISA assay. BV status of samples was assessed using Nugent score. TGase3 was quantified in samples from 14 BV− and 12 BV+ participants. Black bars show standard errors of the measurements. Dots show concentration for individual samples. Star indicates statistically significant difference (Welch’s *T*-test, *P* < .05).

Among the human proteins we identified in our metaproteomic data, 13 that play a role in epithelial structure had a significantly lower abundance in BV+ samples (Table 1). We observed the human protease neutrophil elastase in both BV+ and BV− samples, but its relative abundance was not different based on BV status (Supplementary Table S6). In contrast, the protein leukocyte elastase inhibitor had significantly lower abundance in BV+ samples, indicating that host proteases may be dysregulated in BV. Although many host proteins had a lower abundance in BV+ samples, transglutaminase 3 (TGase3) had a higher relative abundance (Table 1). TGase3 plays a role in epithelial cornification by cross-linking proteins and can also promote wound repair by stabilizing the extracellular matrix [70, 71]. To the best of our knowledge, no previous studies have noted a role for TGase3 in the host response to BV, so we measured the concentration of TGase3 in a separate set of samples to confirm or refute this metaproteomic data. Using an ELISA, we measured the concentration of TGase3 in 26 CVF samples that were not previously analyzed by metaproteomics (14 BV−, 12 BV+). In agreement with the metaproteomic data, we detected TGase3 in all 26 samples and found that its concentration was approximately 2× higher in CVF from BV+ participants (Welch’s *T*-test, *P* = .04) (Fig. 4).

### Signs of heme and iron sequestration in bacterial vaginosis

Iron is a vital nutrient for many pathogenic bacteria, and is required for the *Gardnerella* to grow [72, 73]. In our samples, we identified spectra-matching iron-dependent pyruvate:ferredoxin oxidoreductase from a wide range of BVAB, suggesting that iron is important for organisms including *Prevotella, Dialister,* and *Megasphaera* (Table 2). We also identified a large number of spectra matching the human iron-binding proteins serotransferrin and lactotransferrin, but the abundances of these proteins were not significantly different based on BV status (Supplementary Table S6). Similarly, none of the seven hemoglobin subunit proteins we identified were significantly differentially abundant. However, the heme-sequestering protein hemopexin was present at a significantly higher abundance in BV+ samples and heme-degrading heme oxygenase 1 had a significantly lower abundance (Table 1) [74, 75], suggesting that the host may respond to BV by sequestering heme and reducing its breakdown and the concomitant release of free iron.

## Discussion

In this study, we leveraged metaproteomics to elucidate clinically relevant bacterial processes in BV and associate them with specific taxa. We implicated *D. micraerophilus* in the production of the characteristic BV metabolite putrescine and found that the biosynthesis of this malodorous compound can be increased by inter-species syntrophy between this organism and *F. vaginae.* This interaction likely relies on cross-feeding of ornithine, which is produced as a byproduct of arginine catabolism by the latter organism. Multiple malodorous amines including TMA, cadaverine, and putrescine are known to be present at higher concentrations in vaginal fluid from people with BV [9, 27, 51], but it was not previously known what organisms produce these metabolites. Our results indicate that *D. micraerophilus* is capable of secreting relatively high concentrations of cadaverine in mono-culture. In addition, supplementing this bacterium’s culture media with ornithine or co-culturing it with *F. vaginae* leads to increased putrescine production. Our results also indicate that synthesis of these two polyamines may be co-regulated, because supplemental ornithine led to the total abrogation of cadaverine production. We did not perform genetic knockout experiments to mechanistically confirm *D. micraerophilus* uses ODC to make putrescine, but our findings that this organism secretes malodorous amines could be relevant to other pathologies associated with these compounds such as chronic wounds [76] where *Dialister* spp. have been found [77]. In these polymicrobial infections, related taxa may work with other anaerobic bacteria to synthesize malodorous polyamines.

It remains unclear what biological function putrescine biosynthesis serves for BVAB. Catabolizing arginine into ornithine releases ammonia and produces ATP, but converting ornithine into putrescine does not produce energy. Immune modulation may be one beneficial function for the BV microbiota because putrescine suppresses the inflammatory functions of white blood cells [78]. Alternatively, putrescine’s alkalinity may buffer the pH of vaginal fluid, making it more amenable to acid-sensitive BVAB [79, 80]. Putrescine is also known to inhibit the cross-linking activity of TGase3, slowing the wound-healing process [81, 82]. BV is strongly associated with a disrupted epithelial barrier [83–85], so putrescine may exacerbate damage done by dysregulated host and bacterial proteases. Therefore, targeting this pathway could inhibit multiple functions important for the BV microbiota and ameliorate BV-associated malodor.

Our metaproteomic data suggest *P. micra* and *F. magna* could also contribute to BV malodor by synthesizing TMA from betaine. *Mobiluncus* spp. are currently thought to be the primary producers of TMA in BV because they can produce TMA by reducing trimethylamine *N*-oxide using the enzyme TorA [86]. No studies have identified this enzyme *in vivo*, however, and of the 345 peptide spectra we identified that matched *Mobiluncus* proteins, none were associated with TorA. Instead, we found 29 spectra that matched the TMA-producing enzyme GrdH associated with *P. micra* and *F. magna*. GrdH releases TMA from betaine, which is consistent with Srinivasan *et al.*’s findings that betaine is present in vaginal fluid and has significantly lower concentrations in BV [51].

We identified other proteins in our samples that provided insights into bacterial functions, including extracellular glycoside hydrolases from *L. crispatus* in BV− samples and similar enzymes from *Gardnerella* in BV+ samples. Past studies suggested that human amylases were primarily responsible for releasing metabolizable sugars from vaginal glycogen [87], but our data add to the growing body of evidence that vaginal bacteria are active in glycogen breakdown [50, 88–90]. We also found a higher abundance of bacterial glycosidases in BV+ samples, suggesting that competition for carbohydrates may be elevated in BV, which agrees with past reports of lowered carbohydrate concentrations associated with the condition [51, 91]. Our results also suggest that microbiota is more metabolically active in BV, which is concordant with our qPCR data and other studies that have found higher concentrations of bacteria in individuals with BV [92, 93].

The metaproteomic data provided insights into bacterial fermentation, showing that PFLs are expressed in the vagina by up to 16 genera of BVAB. This observation was unexpected because formic acid and lactic acid have similar pKa values, and BVAB are generally more pH-sensitive than commensal lactobacilli [94, 95]; however, we subsequently confirmed that 12 species of BVAB can produce high concentrations of formic acid *in vitro*. We also confirmed that hypophosphite—a PFL inhibitor and food additive that is generally regarded as safe by the US Food and Drug Administration [96]—severely inhibits formic acid secretion by diverse BVAB and has a stronger antimicrobial effect on formate-producing BVAB between 1 and 10 mM than *L. crispatus, L. gasseri,* and *L. jensenii*. Although these results suggested that formic acid fermentation could be widespread in the BV microbiota, only 4 of 60 CVF samples we tested had detectable concentrations of formate (>130 nM). These measurements were in line with a previous proton nuclear magnetic resonance (^1^H-NMR) study, which confirmed that formic acid is elevated in CVL from individuals with BV, but present at low concentrations (34 nM in BV−, 450 nM in BV+) [97]. These *in vivo* results imply that if bacteria in the BV microbiota secrete formic acid, most of it is quickly consumed. Our results implicate *Coriobacteriales* bacterium DNF00809 as one of the organisms that could perform this function. This fastidious, slow-growing bacterium is highly prevalent in BV [5], and its genome contains homologs of FDH enzymes, which may allow it to metabolize formate for ATP generation [98]. Indeed, DNF00809 grew more efficiently in the presence of formate and depleted both exogenously added and BVAB-secreted formate from its culture media. DNF00809 also significantly increased the pH of spent *G. vaginalis* media. Although it is unclear whether DNF00809 reduced the acidity of spent media primarily by depleting formic acid or through other behaviors such as NH_4_^+^ secretion, these results raise the possibility that minority taxa could play an important role in BV by consuming organic acids secreted by higher abundance taxa such as *Gardnerella* or *Sneathia,* thereby buffering the environment and allowing other bacteria to metabolize more host-derived nutrients. Additional research will be required to determine how prevalent formic acid fermentation is among BVAB *in vivo* and whether other FDH-encoding organisms such as *F. magna* can fulfill a similar function.

The human proteins in our dataset provide insights into potential host responses to BV. Although the iron-binding proteins serotransferrin and lactotransferrin are known to play a role in innate immune defense by sequestering iron [72], we did not observe significant differences in either of these proteins. Two heme-associated proteins were differentially abundant, however. The heme-sequestering protein hemopexin [74] was present at a higher relative abundance in BV+ samples. In contrast, heme oxygenase 1 was present at a lower relative abundance in BV+ samples. Heme oxygenase 1 degrades heme, releasing iron [75]. Together, these data imply that the host may not respond to BV by significantly changing expression of iron-binding transferrins, but may instead respond by sequestering heme away from bacteria and reducing the breakdown of this molecule along with the release of free iron.

A strength of metaproteomic analysis is that proteins are sampled and identified from a community in its natural state; therefore, inferences can be drawn about microbial functions in their normal biological context. This is especially useful in the context of the vaginal microbiota where the dearth of representative model systems makes it difficult to experimentally manipulate human-associated microorganisms [99]. These insights are only inferential, however, and must be complemented with rigorous experimentation to draw conclusions. Our *in vitro* culture experiments supported our hypotheses, based on metaproteomic data, that diverse BVAB secrete formate and a syntrophic relationship could exist between *D. micraerophilus* and *F. vaginae* to produce putrescine. However, these data provide evidence that bacteria are capable of these functions, not that they necessarily perform them *in vivo*. Further studies which leverage systems that better mimic the vaginal microenvironment—such as biofilm flow systems or *ex vivo* organotypic models—are required to better elucidate how formate metabolism and putrescine biosynthesis contribute to bacterial colonization of the vagina.

This study demonstrates the value of analyzing metaproteomic data at different scales. Broad, high-level analysis enables the identification of general functional differences between microbial communities, whereas parsing individual protein identifications can uncover biologically relevant functions performed by different organisms. We identified signs of increased, diversified bacterial metabolism in BV, along with potential host responses to epithelial barrier damage. The bacterial proteins we identified provide evidence for which taxa are responsible for characteristic BV metabolites such as acetate, succinate, and TMA, and suggest cross-feeding interactions based on glutamate. Besides glycolytic enzymes, the most abundant bacterial peptide identified in BV samples was linked to formate metabolism, revealing an unexpected and wide-spread metabolic pathway in BVAB. Further experiments revealed that *Coriobacteriales* bacterium DNF00809 is capable of metabolizing formic acid produced by other BVAB. We also discovered a new, syntrophic interaction between *D. micraerophilus* and *F. vaginae* that can increase putrescine biosynthesis. These proteins represent targets for novel treatments that could address BV symptoms and disrupt metabolic pathways that are critical to the BV microbiota. For example, broad-spectrum protease inhibitors may reduce the activity of both host and bacterial proteases, starving BVAB of amino acids. More targeted interventions such as small-molecule inhibitors of GrdH and ODC could reduce the synthesis of TMA and putrescine and alleviate malodor. PFLs are an especially promising target because these enzymes are encoded by a wide range of BVAB but are completely absent from commensal lactobacilli. We showed that 10 mM of the PFL-inhibitor sodium hypophosphite severely reduced bacterial formic acid secretion and exerted a stronger antimicrobial effect on formate-producing BVAB such as *Gardnerella, Sneathia,* and *Amygdalobacter* than most commensal lactobacilli. Specifically designed small-molecule inhibitors of these enzymes could more efficiently disrupt carbohydrate metabolism in BVAB without harming commensal lactobacilli. Such interventions could supplement or replace existing BV treatments, reduce recurrences, and prevent BV in those at highest risk.

## Supplementary Material

Metaproteomics_Figure_S1_wraf055

Metaproteomics_Figure_S2_wraf055

Metaproteomics_Figure_S3_wraf055

Supplemental_Information_BV_Metaproteomics_S6_wraf055

Table_S1_wraf055

Table_S2_wraf055

Table_S3_wraf055

Table_S4_Polyamine_Peak_Areas_wraf055

Table_S5_CVL_Formate_Metadata_wraf055

Table_S6_Not_Significant_Human_Proteins_wraf055

## Data Availability

Protein sequence database files, metaproteomic search result files, and genes identified by shotgun metagenomic sequencing used in this study are available at 10.6084/m9.figshare.25705839.v1. Code files used for data analysis and figure generation are available at 10.5281/zenodo.11075582. Raw mass spectrometry data generated in this study have been deposited to the ProteomeXchange Consortium via the PRIDE [100] partner repository with the database identifier PXD051980.
