# Supplementary figures and images for: Syntrophic bacterial and host–microbe interactions in bacterial vaginosis
Source: ISME J. 2025 Jun 27;19(1):wraf055. doi: 10.1093/ismejo/wraf055 (PMC12208373; doi:10.1093/ismejo/wraf055)

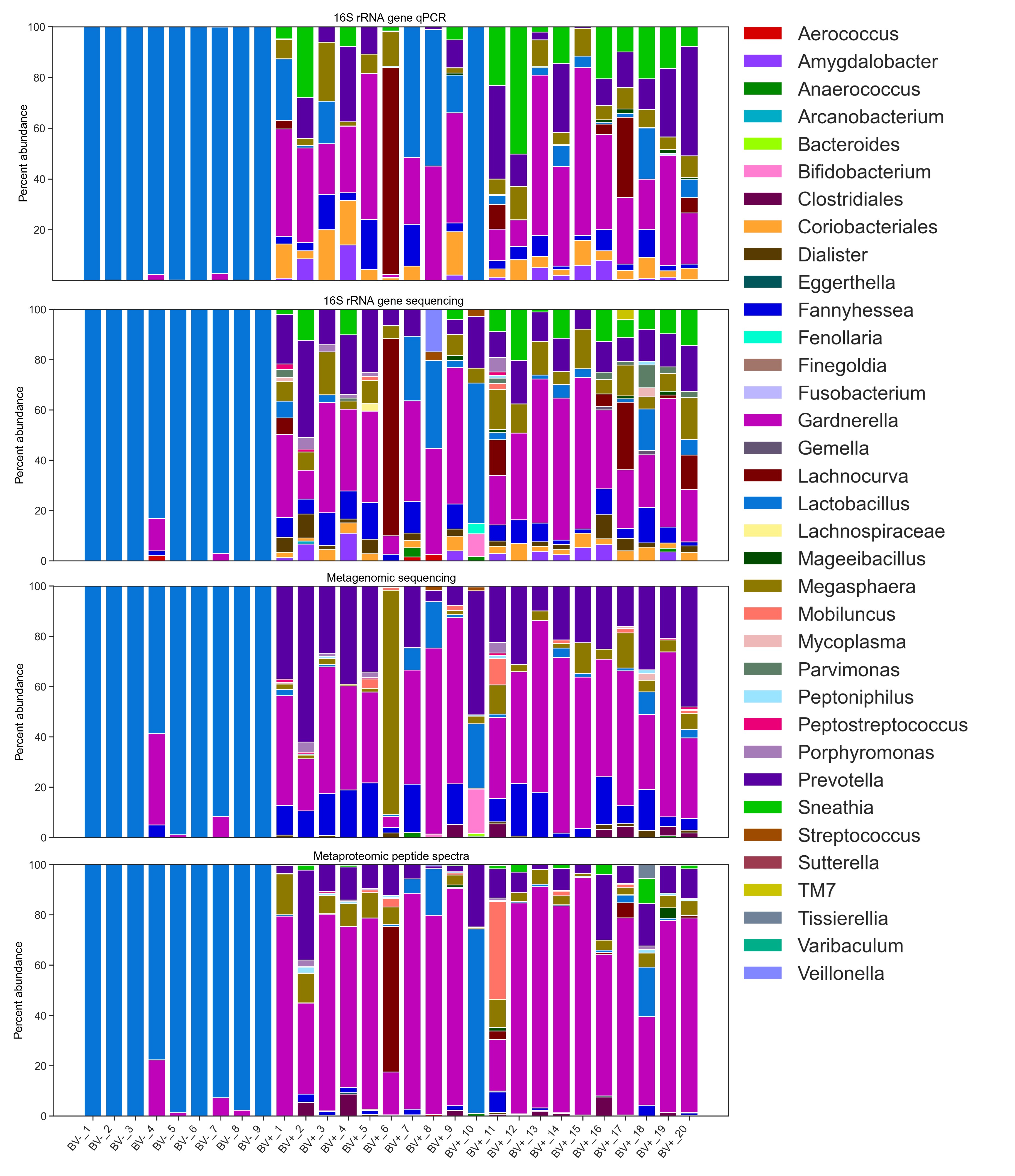

Supplement: Metaproteomics_Figure_S1_wraf055 [file metaproteomics_figure_s1_wraf055.jpeg]

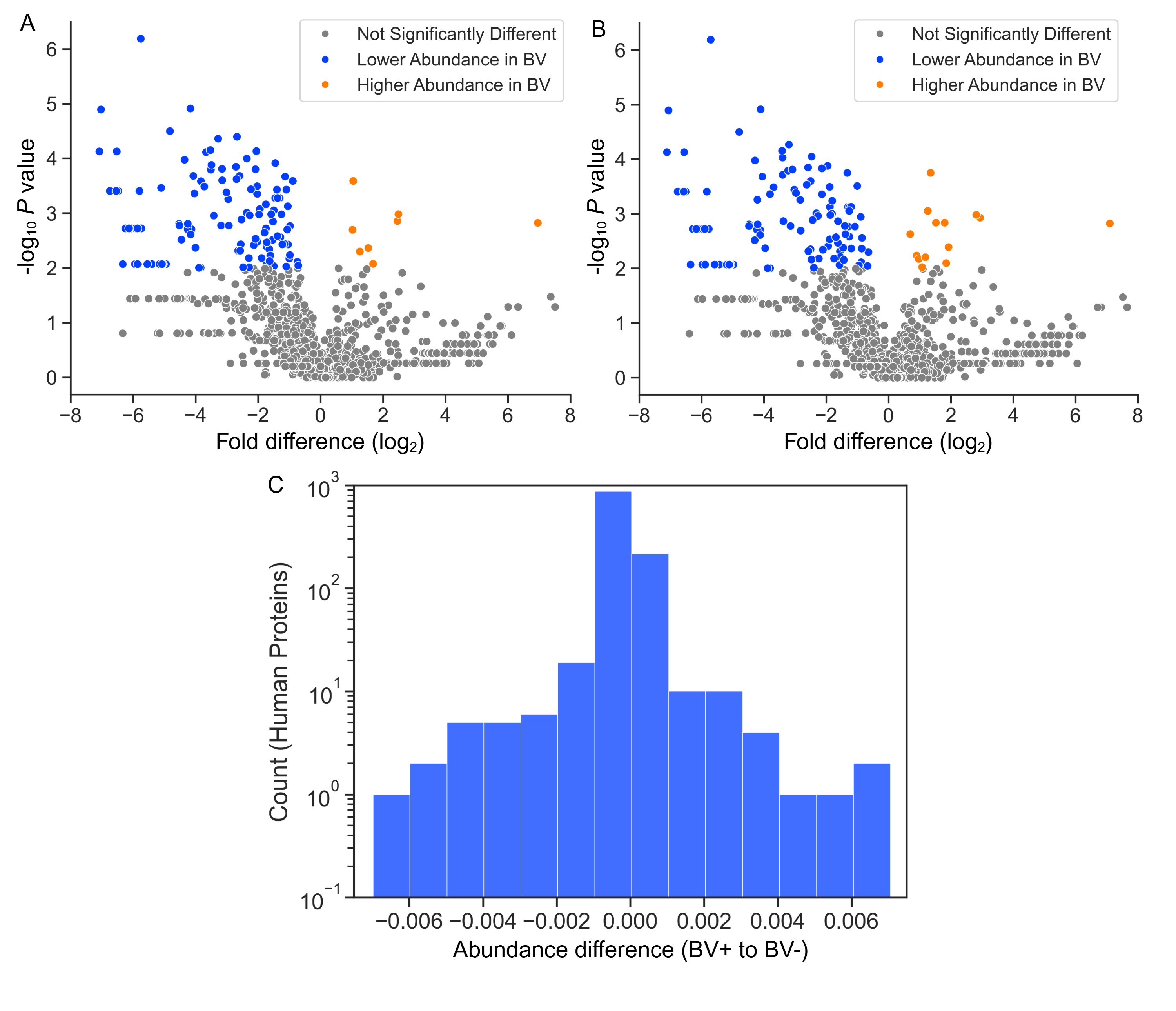

Supplement: Metaproteomics_Figure_S2_wraf055 [file metaproteomics_figure_s2_wraf055.jpeg]

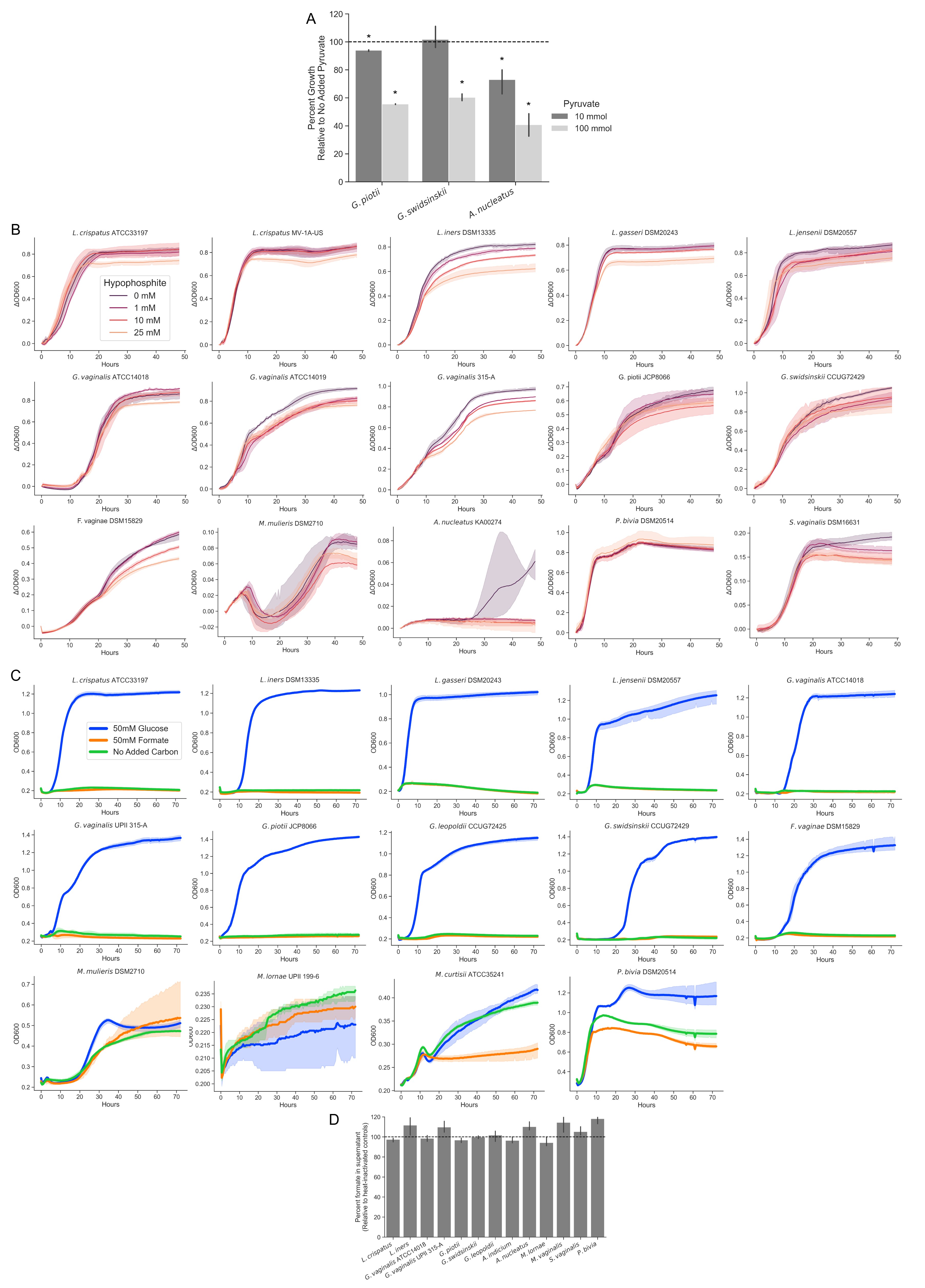

Supplement: Metaproteomics_Figure_S3_wraf055 [file metaproteomics_figure_s3_wraf055.jpeg]
