# Supplementary material for: Syntrophic bacterial and host–microbe interactions in bacterial vaginosis
Source: ISME J. 2025 Jun 27;19(1):wraf055. doi: 10.1093/ismejo/wraf055 (PMC12208373; doi:10.1093/ismejo/wraf055)
Supplement: Supplemental_Information_BV_Metaproteomics_S6_wraf055 [file supplemental_information_bv_metaproteomics_s6_wraf055.pdf]

SUPPLEMENTAL FIGURES

Figure S1. Taxonomic makeup of samples by different analysis methods.
Abundance of bacterial taxa in study samples as determine by species-specific qPCR, broad-range 16S rRNA gene sequencing, metagenomic sequencing, and metaproteomic peptide spectra. Peptide spectra that could not be assigned to a single genus were excluded from this analysis.

Figure S2. Comparison of protein abundance normalization methods.
When calculating the abundance of human proteins in our samples, we tested normalizing their PSMs in a sample to A) the combined number of human and bacterial PSMs in the sample or B) only the number of human PSMs in the sample. Taking the former approach biased our analysis by identifying more proteins as lower abundance in BV+ samples (Total proteome normalization: 452 human proteins higher abundance and 708 lower abundance in BV. Human proteome normalization: 449 human proteins higher abundance and 661 lower abundance in BV) because there were significantly fewer human PSMs in BV+ samples. C) Abundance differences for human proteins by calculating abundance relative to human proteome had a normal distribution, showing that this approach did not skew results.

Figure S3. Formate metabolism by vaginal bacteria.
A) Isolates of *G. piotii, G. swidsinskii,* and *A. nucleatus* were grown in their preferred media on a 96 well plate with added sodium pyruvate proportional to 0, 10, or 100 mmol sodium pyruvate anaerobically at 37°C for 72hrs. Change in OD600 was determined for each well. Four biological replicates were performed for each media type. Wells with added sodium pyruvate were normalized against a paired well without additional pyruvate. The dotted line shows 100% growth where growth was equivalent to the control condition. Bars represent standard error of four measurements. Stars show treatments where bacterial growth was significantly lower compared to no added pyruvate as determined by Mann-Whitney U Test ($P < 0.05$). B) Growth curves for bacteria grown in the presence of different concentrations of sodium hypophosphite. Light-colored bands show 95% confidence interval. ΔOD600 was calculated by subtracting the initial OD600 measurement from measurements at each subsequent timepoint. C) Growth of bacteria in their preferred media with glucose (blue), sodium formate substituted for glucose (orange), or neither glucose nor formate (green) in anaerobic conditions at 37°C for 72hrs. Light-colored bands show 95% confidence interval for three biological replicates. No isolate achieved significantly higher density in the sodium formate media compared to the neither glucose or sodium formate control media. (unpaired T-test, $P > 0.05$). D) Depletion of formate from culture media by different bacteria. Isolates were inoculated into media containing 50mM sodium formate and incubated anaerobically at 37°C for 48hrs. An enzyme-based assay was used to quantify the concentration of formate in the culture supernatant following the incubation. This concentration was then normalized to the amount of formate present in a sample of the same bacteria that had been heat-inactivated at 70°C for 45min, then incubated

alongside the live samples. Black bars show standard error of three separate cultures for each isolate. The dotted line shows 100% formate concentration compared to the heat-inactivated control, below which would indicate the isolate is depleting formate from its media.

SUPPLEMENTAL TABLES

Table S1. Characteristics of study participants for metaproteomic analysis.
[a]Bacterial vaginosis was diagnosed using Amsel clinical criteria.
[b]*N* indicates the number of participants.
[c]The sample from one of the ten BV- participants was contaminated with an unknown polymer which compromised metaproteomic analysis. Thus, this sample was excluded from further analysis and only nine BV- samples were considered.
[d]Other races included Asian/Filipino (one participant) and Native Hawaiian/Pacific Islander (one participant). One participant chose not to specify their race.

Table S2. Human proteins identified in CVL samples.
Identified human proteins are listed with their average spectral count and average log-2 transformed abundance in BV- (N=9) and BV+ (N=20) samples. For analysis, the spectral count of a protein was divided by the total number of spectra matching human proteins in the sample to calculate a relative abundance for the protein. The relative abundance was then log-2 transformed and transformed relative abundances were compared between BV- and BV+ samples by Mann-Whitney U test. *P* values for Mann-Whitney U tests are listed for each identified protein.

Table S3. Bacterial proteins identified in CVL samples.
Identified bacterial proteins are listed with their annotation, all taxa associated with proteins with an identical amino acid sequence, total spectral count in BV- (N=9) and BV+ (N=20) samples, and their total spectral count across all 29 samples.

Table S4. Raw peak areas from targeted polyamine LC-MS analysis of bacterial culture supernatants.
Measured MS peaks for putrescine, cadaverine, and spermidine in positive controls, media, and pooled culture supernatants of *D. micraerophilus* and *F. vaginae.* Spermine was not detected in samples so it was not included in positive controls. N/A: The given polyamine was not detected in the sample.

Table S5. Formic acid concentrations in CVL.

Formic acid was measured in study participant CVL by enzymatic assay. BDL: Below detection limit. Limit of detection for the assay was 0.128mM formate. Date of sample collection, as well as participant BV status on the day of sampling by both Amsel criteria and Nugent score, are also listed for each sample.

Table S6. Biologically relevant host proteins identified in CVL samples

Host proteins identified in CVL samples which were not significantly differentially abundant by BV status. *P* values for Mann-Whitney U tests of each protein's transformed relative abundance between samples from BV- and BV+ participants are shown, along with the average spectral count for each protein in samples from BV- and BV+ participants. The functional category for each protein is also listed.
